# Understanding the structural and functional properties of carbohydrate esterases with a special focus on hemicellulose deacetylating acetyl xylan esterases

**DOI:** 10.1080/21501203.2018.1492979

**Published:** 2018-07-04

**Authors:** Ayyappa Kumar Sista Kameshwar, Wensheng Qin

**Affiliations:** Department of Biology, Lakehead University, Thunder Bay, Ontario, Canada

**Keywords:** Carbohydrate esterases, CAZy, hemicellulose, acetyl xylan esterase, biofuel, animal feedstock

## Abstract

Acetyl and methyl esterifications are two major naturally found substitutions in the plant cell-wall polysaccharides. The non-cellulosic plant cell-wall polysaccharides such as pectin and hemicellulose are differentially esterified by the *O-*acetyl and methyl groups to cease the action of various hydrolytic enzymes secreted by different fungi and bacterial species. Thus, microorganisms have emerged with a special class of enzymes known as carbohydrate esterases (CE). The CE catalyse O-de, N-deacetylation of acetylated saccharide residues (esters or amides, where sugars play the role of alcohol/amine/acid). Carbohydrate active enzyme (CAZy) database has classified CE into 16 classes, of which hemicellulose deacetylating CE were grouped into eight classes (CE-1 to CE-7 and CE-16). Various plant biomass degrading fungi and bacteria secretes acetyl xylan esterases (AcXE); however, these enzymes exhibit varied substrate specificities. AcXE and xylanases-coupled pretreatment methods exhibit significant applications, such as enhancing animal feedstock, baking industry, production of food additives, paper and pulp, xylitol production and biorefinery-based industries, respectively. Thus, understanding the structural and functional properties of acetyl xylan esterase will significantly aid in developing the efficient AcXE with wide range of industrial applications.

## 1. Introduction

Plants have developed an efficient system to guard against invading microorganisms. Acetylation of plant polysaccharides plays a crucial role in protecting plant cell wall from microbial hydrolytic enzymes. Naturally, two types of esterifications such as *O*-acetyl and methyl are observed on plant polysaccharides. Compared to *O*-acetylation, methyl esterification of plant polysaccharides was highly studied. *O*-acetylation is observed in the branches and backbone structures of various cell wall polymers with the nature, and extent of the acetylation significantly varies based on the species, type of tissues and cell walls (Gille and Pauly 2007; Pawar et al. 2013). The sources of *O*-acetyl groups in primary and secondary cell walls of woody plants such as softwood and hardwood are observed to be as homogalacturonan, rhamnogalacturonan I, rhamnogalacturonan II (Pectin), xyloglucan, glucuronoarabinoxylan (type II primary cell walls of grasses), glucuronoxylan and glucomannans (Table 1). The degree of acetylation varies based on the type of tissue and plant species; it was reported that the degree of acetylation varied between 0.60 and 0.75 in glucuronoxylan of aspen wood and 0.3 and 0.4 in galactoglucomannan of spruce, aspen and birch wood (Teleman et al. 2000, 2003). The acetyl groups were also found to migrate between the neighbouring free hydroxyl groups and the positions of which varies based on the species (Pauly 1999; Jacobs et al. 2002; Mastihubová and Biely 2004) (Table 1).

**Table 1.** Structural properties and occurrence of different O-acetylated hemicellulose structures reported below were retrieved from Pawar et al. (2013).

Acetylated forms of polysaccharides	Properties and Occurrence
**(Galacto) Xyloglucan**	Xyloglucans are major constituents of hemicelluloses; xyloglucans contain a backbone of 300 to 3000 β-(1→4)-linked D-glucopyranose residues. 60–75% (in grasses 30–40%) glucose residues have side chains at position 6, which are: D-xylopyranosyl-α-1 →, D-galactopyranosyl-β-(1→2)-D-xylopyranosyl-α-I→, L-arabinofuranosyl-(1→2)-D-xylopyranosyl-α-1 →, and (except in grasses) L.-fucopyranosyl-α-(1 -→2)-D-galactopyranosyl-β-(1-→2)-D-xylopyranosyl-α-1 →) (Fry 1989).
**Galactoglucomannan**	Galactoglucomannan is group of hemicelluloses soluble in water containing simple sugars, such as D-galactose, D-glucose and D-mannose. Structurally, galactoglucomannan contains a backbone of (1→4) linked D-mannose units with (1→6) D-galactose units, with hydroxyl groups in C-2, C-3 positions of mannose are substituted with acetyl groups (Willför et al. 2008).
**Glucuronoxylan**	Glucuronoxylan is one of the major components of hemicelluloses in hardwoods (8.9% in Pine and 27.5 Birch wood). Structurally, glucuronoxylan contains glucuronic acid and xylose, forming linear polymers of β-D-xylopyranosyl units linked through (1→4) glycosidic bonds.(Kibblewhite and Brookes 1976; Mitikka et al. 1995)

### 1.1 Properties of hemicellulose and microbial approach to degrade it

Hemicellulose, a heterogeneous polysaccharide containing xylans, mannans, arabinans, galactans, glucuronoxylans, arabinoxylans, glucomannans, β-(1→3,1→4)-glucans and xyloglucans thus is difficult to be separated in its pure form of monomeric sugar (Scheller and Ulvskov 2010). However, mechanisms behind the biosynthesis of xylans and glucans in the plant cells need to be understood (Scheller and Ulvskov 2010; Dalli and Rakshit 2015). Plant biomass generally contains 40–50% of cellulose, 20–30% of hemicellulose, 15–30% of lignin and 5–10% of pectin, typically this percentage of hemicellulose differs among the plant species (Dalli and Rakshit 2015). Hemicellulose plays a crucial role in strengthening and development of plant cell walls, as it occurs in close association with cellulose and lignin. According to Malunga and Beta (2015), arabinoxylan extracted from wheat and barley was found to contain antioxidant properties, as it acts as a stock for large quantities of ferulic acid and other phenolic compounds (Dalli and Rakshit 2015; Malunga and Beta 2015). Plants synthesise hemicelluloses from nucleotide sugars through glycosyl transferases in the Golgi membranes. Xylans are the major component present in primary walls constituting to about 20%, majority of the xylans are acetylated (O-3 and O-2 positions of xylose residues). Apart from xylans, mannans and glucomannans are also acetylated in the plant cell walls. In hardwood xylopyranosyl residues and in softwood glucuronoxylan and mannopyranosyl residues of hemicellulose are generally acetylated at O-3 and O-2 positions (Biely 2012).

It was already known that acyl groups present on the plant polysaccharides change their physicochemical properties and hinder the process of hydrolysis through glycoside hydrolases. The weak acid nature of these acetylated polysaccharides along with the furan and phenolic derivatives of the plant cell wall results in reduced rate of hydrolysis and fermentation leading in lesser yields with much lesser productivities (Carvalheiro et al. 2008). Earlier studies have proved that alkaline pretreatment of plant biomass eases the process of enzymatic degradation and digestion in animals (Bacon et al. 1975; Chesson et al. 1983; Biely 2012). According to Biely (2012), alkaline pretreatment saponifies the alkali-labile ester linkages among the plant cell-wall components and loosens the intricate structure through breaking down the physicochemical bonds linking these cell wall materials (Biely 2012).

Microorganisms secrete a wide range of glycoside hydrolases for the breakdown of cellulose and hemicellulose. The acetylated glycosyl residues of plant polysaccharides (*O-*acetylated) prevent the action of glycoside hydrolases to break the glycosidic linkages. Thus, microorganisms have come up with a special class of enzymes known as carbohydrate esterases (CE) to de-acetylate hemicellulose and pectin units of plant polysaccharides. The CAZy database has classified the CE into 16 different classes (CE-1 to CE-16) (Table 2) (Lombard et al. 2014). CE exhibit a wide diversity in substrate specificity and structural properties. Based on their substrate specificity, the enzymes in 16 classes of CE can be mentioned as acetyl xylan esterases (AcXE), acetyl esterases, chitin deacetylases, peptidoglycan deacetylases, feruloyl esterases, pectin acetyl esterases, pectin methylesterases and glucuronoyl esterases (Biely 2012). Majorly CE can be divided into two categories: hemicellulose deacetylating and pectin deacetylating CE. The CE enzymes belonging to CE-1 to CE-7 and CE-16 classes can be classified as hemicellulose deacetylating CE; similarly, pectin deacetylating CE belongs to CE-8 and CE-12 classes.

**Table 2.** Different classes of carbohydrate esterase (CE) family and their corresponding representing enzymes with note on their protein 3-D structure.

CE- Class	Representing Enzymes	E.C. Number	3D Structure Status
CE-1	**acetyl xylan esterase**, cinnamoyl esterase, feruloyl esterase, carboxylesterase, S-formylglutathione hydrolase, diacylglycerol O-acyltransferase, trehalose 6-O-mycolyltransferase	(EC 3.1.1.72) (EC 3.1.1.-) (EC 3.1.1.73) (EC 3.1.1.1) (EC 3.1.2.12) (EC 2.3.1.20) (EC 2.3.1.122)	(α/β/α)-sandwich
CE-2	**acetyl xylan esterase**	(EC 3.1.1.72)	α/β + β-sheet
CE-3	**acetyl xylan esterase**	(EC 3.1.1.72)	(α/β/α)-sandwich
CE-4	**acetyl xylan esterase**, chitin deacetylase, chitooligosaccharide deacetylase, peptidoglycan GlcNAc deacetylase, peptidoglycan N-acetylmuramic acid deacetylase	(EC 3.1.1.72) (EC 3.5.1.41) (EC 3.5.1.-) (EC 3.5.1.-) (EC 3.5.1.-)	(β/α) 7 barrel
CE-5	**acetyl xylan esterase**, cutinase,	(EC 3.1.1.72)	(α/β/α)-sandwich
CE-6	**acetyl xylan esterase**	(EC 3.1.1.72)	(α/β/α)-sandwich
CE-7	**acetyl xylan esterase**, Cephalosporin-C deacetylase	(EC 3.1.1.72) (EC 3.1.1.41)	(α/β/α)-sandwich

The Europian Union report  (2007) has extensively reported the potential market value of hemicellulose worldwide with total of 153.5 million £ from USA, China, Switzerland, Argentina, Japan and Europe alone can generate 848.9 million £ (Wysokińska 2010). These reports suggest that hemicellulose can be highly valuable potential product, which can generate large revenues (Dalli and Rakshit 2015). To achieve these great revenues, it is necessary to separate these polysaccharides into almost pure forms as oligosaccharides or monosaccharide to produce great yields of bioproducts, which can only be achieved through developing a consolidated biorefinery industries with abilities for separation, pretreatment, detoxification, fermentation and purification (Dalli and Rakshit 2015). Thus, it is necessary to understand properties of these deacetylating enzymes to facilitate efficient hydrolysis and fermentation by the hydrolytic enzymes. In this article, we have extensively discussed about the structural and functional properties of various CE involved in deacetylation of plant cell-wall polymers.

## 2. Microbial enzymes depolymerisation of hemicellulose

It was well established that complete hemicellulose degradation requires a combination of hemicellulolytic enzymes, such as endo-β-l,4-xylanase and β-xylosidase, and other accessory enzymes, such as α.-arabinofuranosidase, α.-glucuronidase, acetyl xylan esterase and ferulic acid esterases (Saba and Botbast 1999). Zhang et al. (2011). Previous studies have reported that even after the application of hydrothermal, steam explosion pretreatments on plant biomass, most of the substituents such as acetyl residues, might remain intact with the xylan chain and obstruct the action of xylanases during the enzyme hydrolysis (Zhang et al. 2011). Xylan hydrolysis is significantly increased by the removal of acetyl side chains, and the hydrolysis is hindered by the degree of acetylation (Poutanen et al. 1987). Grohmann et al. (1989) have reported that chemical deacetylation of aspen wood and wheat straw xylan units has enhanced the enzymatic hydrolysis of xylan and thus increased the cellulose accessibility (Grohmann et al. 1989).

AcXE are secreted by microorganisms for the deacetylation of xylan polymers and xylooligosaccharides (E.C. 3.1.1.72) (Biely 2012). AcXE are widely distributed among different CE classes (CE-1 to CE-7). Biely et al. (1985) have found the occurrence of acetyl xylan esterase in the cellulolytic and hemicellulolytic microorganisms (Biely et al. 1985). After that several studies have reported the occurrence of the acetyl xylan esterase as they have found to act on acetyl glucuronoxylan; however, later it was found that AcXE is active on other acetylated polysaccharides other than xylan (Biely 2012). The microbial endoxylanases were found to work in synergy with CE, especially the activity of endoxylanases on acetyl xylan increased with the presence of AcXE (Biely et al. 1986). Acetyl xylan (O-acetyl-4-O-methyl-Dglucurono- D-xylan) is the naturally occurring form of hemicellulose in hardwood, alternate xylopyranosyl units of the polymeric xylan contains one acetyl group (Biely et al. 1986; Biely 2012). Selig et al. (2008) and has showed that combinatorial action of endoxylanases and AcXE has significantly improved the hydrolysis of xylan polymer (Selig et al. 2008). Selig et al. (2009) have reported that when AcXEs were used in combination with endoxylanases on different corn stover substrates, a linear relationship in removal of acetyl groups and depolymerisation of xylan was observed (Selig et al. 2009). The hydrolysis of xylan from the pretreated wheat straw and giant reed using xylanolytic enzymes and AcXE was conducted by Zhang et al. (2011). This study has showed clearly that removal of acetyl groups by AcXE enhanced the accessibility of xylan by the xylanolytic enzymes, and solubilisation of xylan has progressively increased the availability of cellulose to cellulases, resulting in hydrolysis of cellulose (Zhang et al. 2011). Cellulolytic and xylanolytic enzymes function in synergistic effect, and the combined use of cellulases, xylanases and AcXE has resulted in higher hydrolysis of cellulose revealing the occurrence of acetylated xylan in the cellulose matrix (Zhang et al. 2011). In this article, we have extensively reviewed the structural and functional properties of AcXE occurring in different CE classes.

### 2.1 Substrate and positional specificity of AcXE

The AcXE are class of enzymes which exhibit higher substrate specificity towards O-acetyl-4-O-methyl-D-glucurono-D-xylan, which can also be called as acetyl xylan occurring in the hardwood hemicellulose. Most AcXEs universally exhibit acetyl esterase activity except AcXEs classified under CE class-4. The CE class-I AcXEs exhibit strong substrate specificity towards acetylgalactoglucomannan and acetylated hexosides, cellulose acetate and other carbohydrate residues, respectively (Biely et al. 1996a; Prates et al. 2001; Schubot et al. 2001; Altaner et al. 2003). The CE class-2 AcXEs are active on hexopyranosyl residues of the oligo- and polysaccharides with lower activity against acetylxylan and xylanoside residues. These enzymes are known for its transesterification reactions of hexose and hexosyl residues specifically on position 6, respectively (Dalrymple et al. 1997; Montanier et al. 2009b; Topakas et al. 2010). In contrast with class-1 and 2 enzymes, CE class-3 AcXEs exhibit substrate specificity towards a wide range of acetylated carbohydrate residues. Studies also report that some of the AcXEs need experimental proofs to support their classification under a specific CE class (Weadge and Clarke 2007; Correia et al. 2008). Unlike other class-1, 2 and 3 CE class enzymes, CE class-4 enzymes are highly specific towards the acetyl xylan substrates and these AcXEs are not active against acetylgalactoglucomannan or acetylated manno-residues. However, the classification of chitin and peptidoglycan deacetylating AcXEs under CE class-4 explains the characteristic ability of CE class-4 AcXEs activity on chitin (Biely et al. 1996b; Caufrier et al. 2003; Blair et al. 2005; Taylor et al. 2006). The CE class-5 AcXEs show their specificity towards acetyl xylan residues, acetylated xylo-oligosaccharide, mannosides and cellulose acetate residues by deacetylating them on position 2. These enzymes are not active on acetylgalactomannan residues (Biely et al. 1997a, 1997b; Hakulinen et al. 2000; Altaner et al. 2003; Tenkanen et al. 2003). The CE class-6 AcXEs are considered as real AcXEs, as they precipitate the acetylxylan from the solution, but reports also suggest that CE class-6 AcXEs exhibit a broad substrate specificity (Blum et al. 1999; Bitto et al. 2005; Krastanova et al. 2005; Lopez-Cortés et al. 2007). The CE class-7 AcXEs exhibit a strong ability to DE acetylate wide range of acetylated carbohydrate substrates; however, studies report that intracellular localisation of the CE class-7 enzymes does not support their classification as AcXE as they do not get to interact with the polymeric substrates. This might propose that these enzymes take part in the degradation of the acetylxylan in its final stages. The CE class-7 AXE’s exhibit a very low positional specificity, these enzymes also exhibit a cephalosporin C deacetylase activity, respectively (Vincent et al. 2003; Kremnický et al. 2004; Krastanova et al. 2005; Biely et al. 2007; Biely 2012; Levisson et al. 2012). The activity of AcXe is affected by the substrate and positional specificity, respectively. The mode of AXE’s action can vary based on their deacetylation patterns of xylopyranosyl residues at the positions 2/3 or at a single position, respectively (Biely 2012). Previous studies conducted by Biely P et al. have reported that double deacetylation of acetylated methyl-β-D-xylopyranosides at positions 2 and 3 by CE class-1, 4 and 5 AcXEs (Biely et al. 1996b, 1997a).

### 2.2 *Penicillium purpurogenum* AcXEII (CE class-i)

X-ray crystallographic studies of *Penicillium purpurogenum* acetyl xylan esterase (AcXEII) revealed that 207 amino acid long AcXEII classified under the new class of α/β hydrolases with substrate specificity towards D-xylopyranose acetate esters occurring in xylan (Pangborn et al. 1996; Ghosh et al. 1999, 2001). Structurally, about 125-amino acids are arranged in 10 β-strands (β1 to β10) and six α-helices (α1 to α6), and the remaining 82 amino acid residues were arranged into five type-1 and type-2 β turns, one γ-haripin turn and five extended loop regions. There are five inter-chain disulphide bridges: Cys2-Cys79, Cys46-Cys55, Cys101-Cys161, Cys147-Cys179 and Cys171-Cys178. Tertiary structure of AcXE II contains doubly wound α/β sandwich with central parallel β-sheet flanked by two α-helices on each side. The exposed region of the molecule located at the C-terminal end of the β-sheet is bordered by helical residues (183–193) on one side and loop with antiparallel pair of strands on the other side (105–113). The overall structure resembles a cylinder with an average diameter and height of 27 (63) Å and 38 (63) Å, respectively. These high-resolution studies conducted by Ghosh et al. (2001) have revealed the coexistence of two different conformational states representing the two different oxidation states of Serine and Histidine (catalytic residues) in the same crystal (Ghosh et al. 2001).

The manual docking of AcXEII by 2-acetyl xylopyranose showed that acetate is at a van der Waal’s contact distance with the backbone atoms Thr^13^ NH and His^187^ CO and side-chain atoms of His^187^, Glu^12^, Tyr^89^ and Gln^188^ resulting in a pocket for accommodating acetate moiety. The biochemical studies conducted earlier also proves the fact that AcXE has strong substrate specificity towards acetate esters, thus can weakly hydrolyse long fatty acid esters (Egana et al. 1996). The amino acid residues Tyr^57^, Gln^91^ and Phe^152^ of the AcXEII come in direct contact with the substrate. The active site gorge (20Å) containing anti-parallel loop λ3 could accommodate about 4 to 5 xylose residues in the xylan chain; these xylose units contact the aminoacid residues Ser^58^, Asp^153^, Glu^158^ and Asn^107^. The residues from 13 to 20 of λ1 segment and residues from 41 to 55 of λ2 segment border the catalytic site gorge to the left, where β3 to α3 turn on the centre residing in the side chains of the three residues. The helix α5 residues from 175 to 183 of λ5 segment, β6 to β7 residues from 132 to 134 and residues from 152 to 161 of λ4 segments are bordered on the right of the active site gorge. Amino acidsresidues Glu12, Tyr89, Ser21, Thr24, Gly191, Tyr190, Thr186, Pro176, Tyr177, Gln188, Thr13, Thr14, Gln49, Gly47, Tyr57, Pro134 and Phe152 line the active site gorge (Ghosh et al. 2001). The sulphate ion molecule, a glycerol moiety and 40 molecules of water occupies the entire catalytic cleft. The anti-parallel loop λ3 containing β4–β5 present in the active site gorge might also function as a recognition and binding site for the xylan chains, through which it acts as a gateway for the binding of xylan chain to the enzyme active site. The loop λ3 is loosely connected to the enzyme structure through hydrogen bonds (Gln^108^ Ne^2^→ Od^1^ Asp^153^ (2.92 Å), Asn^107^ Nd^2^ → OC Phe^152^ (2.99 Å) and Asp^105^ Od1 → Oe^1^ Glu^94^ (2.54 Å)). The existence of protein in two different conformation states (A and B) is one of the important findings revealed by Ghosh et al. (2001) (Ghosh et al. 2001). The amide sidechains of Thr13 and Gln91, hydroxyl groups of Ser90 (Oγ) and Thr13(Oγ) constitutes for the oxyanion binding site for the A conformation state of the protein. The oxygen atom of the water (598) molecule binds at the oxyanion binding site in tetrahedral geometry and Thr13 side chains are stabilised by Nε2 from Gln91 by hydrogen bonding. However, in the B conformation state, 598th water molecule is replaced by SO_4_ (210), as O3 group of the sulphate coincides with the oxygen group of water molecule (Ghosh et al. 2001).

**Figure 1.**
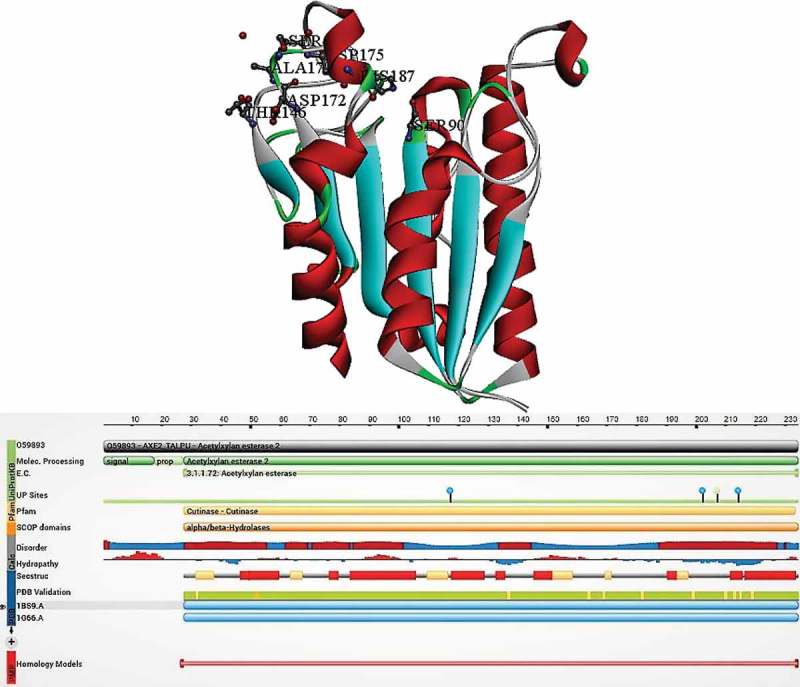
Structural properties of *Penicillium purpurogenum* acetyl xylan esterase (carbohydrate esterase class-I) (PDB ID:1BS9) (Ghosh et al. 2001).

The structural properties of AcXEII make it a pure esterase and not a lipase, which acts on long fatty acid ester chains. The following structural properties prove the openness and exposed nature of AcXEII active site gorge the following: a) the availability of catalytic residues Ser and His is high for AcXEII, with a water molecule acting as probe; b) Along with Ser^90^ and His ^187^, the side chains of AcXEII are uncovered; c) Amino acid residues, Tyr^177^, Tyr^57^ and Phe^152^, protect the active site gorge thus decreasing the accessibility; and d) gatekeeper loop (loop λ3) also limits the access of the active site gorge for the xylan chain. The well-arranged secondary structure elements and the disulphide-stabilised loops and turns form the major reason behind the internal firm structure of AcXEII. The fewer hydrophobic and several polar interactions in the AcXEII molecule facilitate the interactions between the solvent molecules and solvent-mediated protein–protein connections, these characteristics forms the monomeric globular proteins (Ghosh et al. 2001) (Figure 1).

### 2.3 *Cellvibrio japonicus* AcXEII (CE class-2)

**Figure 2.**
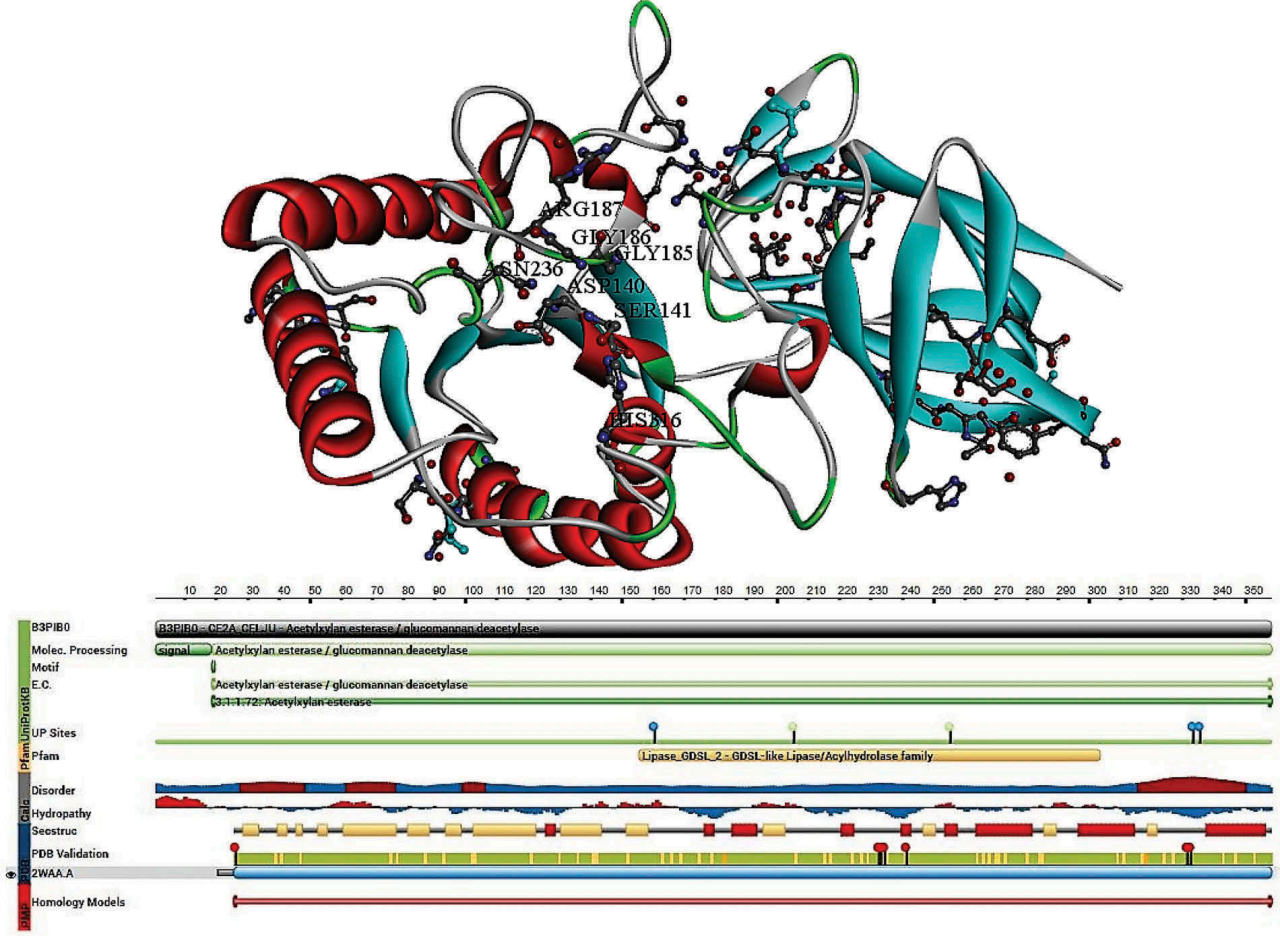
Structural properties of *Cellvibrio japonicus* (carbohydrate esterase class-2) acetyl xylan esterase (PDB ID:2WAA) (Montanier et al. 2009b).

Montanier et al. (2009a) have explained the structural and functional aspects of three variant mature enzyme forms (*CjCE2A, CjCE2B* and *CjCE2C*) of *C. japonicus* (CE class 2) (Montanier et al. 2009a). The three mature forms of *Cellvibrio japonicus* AcXE are bidomain enzymes with about 130 amino acid residues forming an N-terminal β-sheet (jelly roll) domain, which is linked to a 220 amino acid residues C-terminal domain having a typical α/β hydrolase fold (SGNH-hydrolase) (Montanier et al. 2009a). The C-terminal domain consists of β-α-β motifs forming a curved central five stranded parallel β-sheet in the order of β2, β1, β3, β4 and β5 with β2 interrupting the loop insertion. The β-sheet packs against the two α-helices (α1 and α6) on the concave side and three helices (α2, α4 and α5) on the convex side are arranged antiparallel to the β-strands. Along with the above-mentioned helices, *CjCE2* also contains small α-helix, α-3 in the loop is connected by β3 and α4, and 3_10_ helix is present between β1 and α1 structures. Cedric et al. have superimposed the *Clostridium thermocellum* CE class-2 enzyme (*CtCE2*) with *CjCE2A* and *CjCE2B* showing a considerable structural conservation of N-terminal β-sheet domain over 107 and 94 Cα residues with root-mean-square deviations (rmsd) of 1.5 (*CjCE2A)* and 1.9 (*CjCE2B),* respectively. At C-terminal catalytic domain, the rmsd of *CjCE2A* (196 Cα) and *CjCE2B* (195 Cα) also exhibits a substantial structural conservation of *CjCE2A* and *CjCE2B* esterases (Figure 2).

The secondary structure of *CjCE2A* reveals the esterase catalytic centre in the α/β hydrolase of C-terminal, the catalytic triad of *CjCE2A* contains Ser-His-Asp amino acid residues where Ser-160 acts as catalytic nucleophile, His-355 is involved in activation of Ser-160 and Asp-33 connects with Nδ1 of His-335 through hydrogen bond completing the triad structure of catalytic domain. The early transition state of the enzyme stabilised by oxyanion consists of Ser-160 (N), Gly-205 and Asn-255 (Nδ2). The pocket present in the split of *CjCE2A* consists of a formate molecule, which imitates the reactive intermediate that connects with the residues to form the oxyanion hole through hydrogen bonds. When compared to catalytic domain of *CjCE2A, CjCE2B* does not contain Asp residue on its side chain, thus deviating from the classical catalytic triad Ser-His-Asp. However, *CjCE2B* displays a catalytic dyad Ser-His, where histidine (from main chain carbonyl groups) stabilises the catalytic domain. The pull-down assays conducted by Montanier et al. (2009a) have showed that either *CjCE2A* or *CjCE2B* binds to the insoluble cellulose, and CE-2 enzymes of *C. japonicus* are not inhibited by the xylohexaose, cellohexaose and mannohexaose; isothermal titration calorimetry has supported these results as these enzymes does not bind to oligosaccharides (Montanier et al. 2009a). Boraston et al. (2004) have reported that aromatic side chains present in the catalytic sites of proteins play a crucial role in protein carbohydrate recognition (Boraston et al. 2004). As the catalytic core domain of the CE class-2 enzymes is in the cleft which extends towards the catalytic domain, it constitutes to the substrate binding site of CE class-2 enzymes. Thus, to study the substrate binding properties of CE class-2 enzymes, Montanier et al. (2009a) have conducted the site directed mutagenesis of aromatic residues tyrosine (Y665A) and tryptophan (W746A and W790A). Single aromatic residue (Trp-212) mutation in the substrate binding cleft of *CjCE2A* does not affect the *k_cat,_* but lead to the significant increase in *K*_M_ with glucomannan and xylan, which suggest that Trp-212 contributes to the substrate binding (Montanier et al. 2009a).

### 2.4 Clostridium thermocellum and streptomyces lividans (CE class-4)

Taylor et al. (2006) have performed the X-ray crystallography studies on the CE class-4 enzymes from *Clostridium thermocellum (CtCE4)* and *Streptomyces lividans (SlCE4)*, by cloning and expressing the esterase domains of the enzymes as separate entities (Taylor et al. 2006). Mature *SlCE4* CE-4 enzyme consists of 233 amino acid residues forming a bidomain enzyme with C-terminus of the enzyme encodes for CBM2 module (Carbohydrate binding module) with first 41 residues coding for a cleaved signal peptide. The *CtCE4* was reported to be derived from the xylanase (Xyn11A) of *C. thermocellum*, where the C-terminus of the protein contains CE class-4 domain and N-terminus of the protein consists of endoxylanase catalytic domain, CBM6 (Carbohydrate binding module) and a dockerin domain, which is involved in direction of the enzyme to the cellulosome machinery (Carvalho et al. 2003). Both *CtCE4* and *SlCE4* display (β/α)_8_ distorted structures of barrel folds. *SlCE4* is a dimeric structure with a surface area of ~ 2400 Å^2^ and partially occupied Zn^2+^ on the N-terminal of the barrel in a shallow surface groove. The two aromatic residues His-62 and His-66 coordinate the metal ions with Asp-13 and single molecules of water and acetate, thus metal ions were organised in a distorted octahedral arrangement in which acetate ion makes the bidentate. The arrangement of two histidines, an aspartate, gives the CE class-4 enzymes a classical coordination for the bivalent metal ion-dependent hydrolases. The 3-D structure of *SlCE4* possesses high similarity (34% sequence identity) with the peptidoglycan de-N-acetylase (NodB) domain of *Streptococcus pneumoniae* (*SpPgdA*).

Protein structures of *SlCE4* and *SpPgdA* share similarity in their catalytic centres, such as a) amino acid residues present in the active site and substrate binding regions; b) metal ion interactions and acetate molecule positioning; c) aromatic residues (Tyr-103, Trp-124, Trp-131 and Leu-153) which are required for the hydrophobic sheath formation; d) Asp-127 residue which is required for the maintenance of Trp-131 was also found to be conserved among the protein structures of *SlCE4* and *SpPgdA*. The rmsd of *SlCE4* and *SpPgdA* was found to be 1.1 Å. However, these structures can be majorly differentiated based on their protein structures; N-terminus of *SpPgdA* consists two domains and in *SlCE4* C-terminus is involved in the dimerisation of the AcXE. It is reported that *SlCE4* also exhibits a strong activity against chitin by acting as chitooligosaccharide de-N-acetylase (Caufrier et al. 2003).

The *C. thermocellum* CE-4 (*CtCE4*) enzyme is a monomeric protein with 480–684 residues (where 684 refers to C-terminus His6-tag attached to *CtCE4)* part of cellulosome. When *CtCE4* was superimposed with *SlCE4,* a rmsd of 1.2 Å was obtained for 170 equivalent Cα atoms. Although *CtCE4* shares structural similarity with *SlCE4* and *SpPgdA*, it can be differentiated based on the topographical arrangement of substrate binding groove and active site chemistry and metal ion organisation. Structurally, *CtCE4* consists one metal site (Co^2+^, Mg^2+^) both showing an octahedral coordination; in contrast to the *SlCE4, CtCE4* contains only one histidine residue, thus the metal ion coordinates with Asp-488 and His-539 residue and four molecules of water. The changes in coordination results due to Tyr-543 residue, forming an aromatic platform in substrate-binding cleft for binding of ligand. It was reported that these differences might be due to sequences only, as it exists CE class-4 enzymes of *C. thermocellum-*related species, such as *Clostridium cellulovorans* (60% sequence identity) and *Thermotoga maritima* (54% sequence identity). When compared to the topographical structure of *SlCE4, CtCE4* contains Trp-131 but lacks aromatic residue equivalents to Tyr-103 and Trp-124 of *SlCE4*, which might explain *CtCE4* inactivity towards chitooligosaccharides (Taylor et al. 2006).

**Figure 3.**
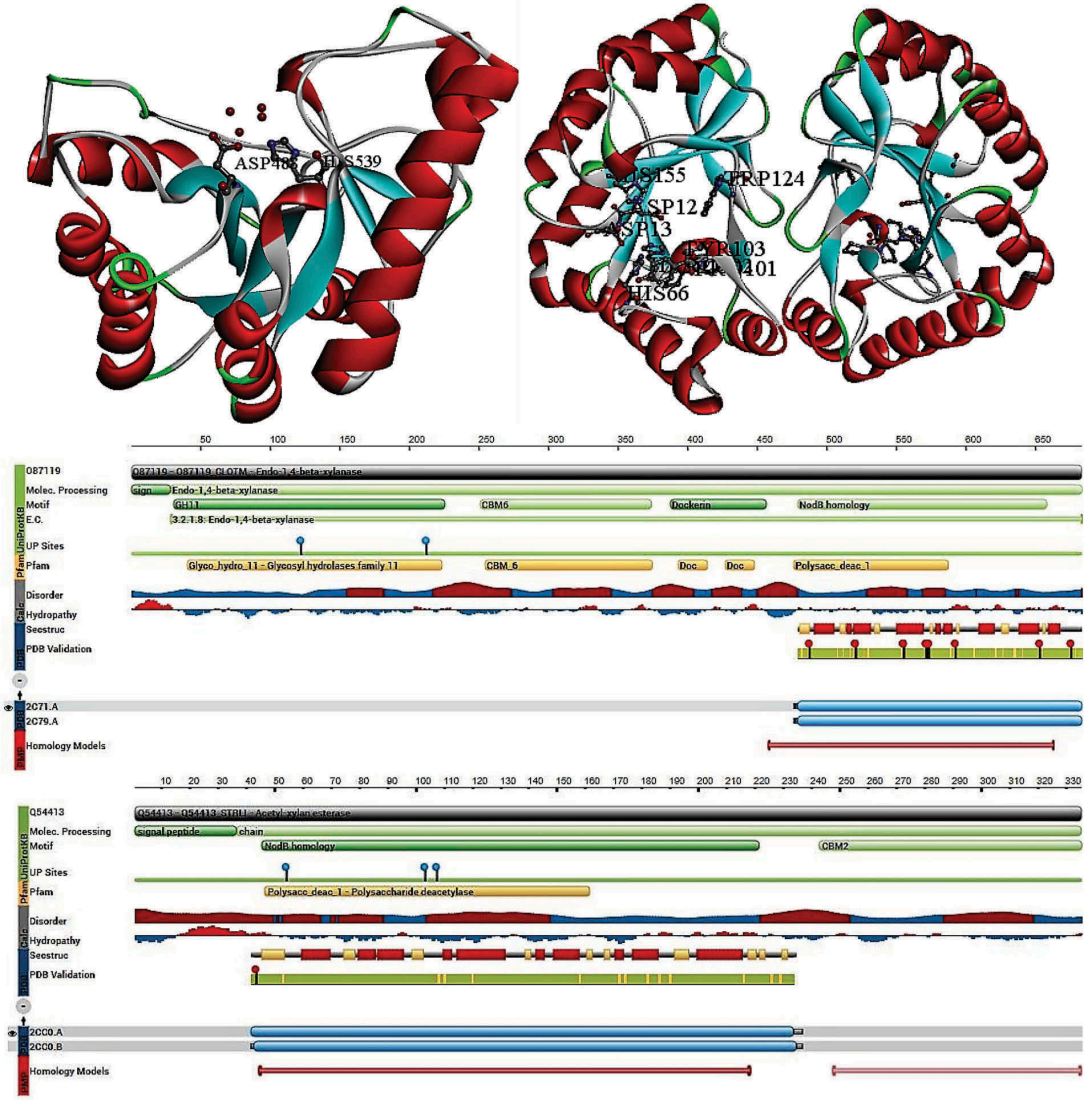
Structural properties of *Clostridium thermocellum* and *Streptomyces lividans* acetyl xylan esterase (carbohydrate esterase class-4) (PDB ID:2C71 and 2CC0), A, C) complete structure of CtCE4; B, D) Topological view of CtCE4 (Taylor et al. 2006).

According to Taylor et al. (2006), both CE class-4 enzymes *SlCE4* and *CtCE4* can be classified as two metal-ion-dependent AcXE (Taylor et al. 2006). *SlCE4* and *CtCE4* can tolerate Mn^2+^ and can exhibit ~ 40–45% maximal activity, with Zn^2+^ metal ion, *SlCE4,* exhibited 33% maximal activity, *CtCE4* showed only 6% maximal activity. Similar difference was observed in *SlCE4* and *CtCE4* with Cd^2+^, *SlCE4* showed 46% maximal activity where *CtCE4* exhibited only 6% maximal activity. Contrastingly, *CtCE4* exhibited better stability and maximal activity with Ni^2+^ and Mg^2+^ with 35% and 3.5%, respectively; whereas *SlCE4* was inactive in the presence of Mg^2+^ and showed 10% maximal activity with Ni^2+^. The Co^2+^ metal is considered as optimal for both the enzymes; however, due to its bioavailability, it does not seem that Co^2+^ is preferred in *in vivo* conditions for the *SlCE4* and *CtCE4* (Taylor et al. 2006). The *SlCE4* and *CtCE4* showed no activity against para-nitrophenyl acetate (standard esterase substrate) as these enzymes possess high specificity towards sugar-based substrates thus these enzymes precipitate acetyl xylan through deacetylation. The metal ion well-arranged between the acetate ion and two histidine gives the *SlCE4* and gives the metal ion Lewis-acid assistance for the nucleophilic attack. The catalytic mechanisms of hydrolases especially Zn^2+^ are highly studied and explained in the past (Hernick and Fierke 2005), it involves base catalysed generation of hydroxide ion, the metal undergoes a direct nucleophilic attack at the carbon centre (Taylor et al. 2006) (Figure 3).

### 2.5 *Trichoderma reesei* AcXEII (CE class-5)

**Figure 4.**
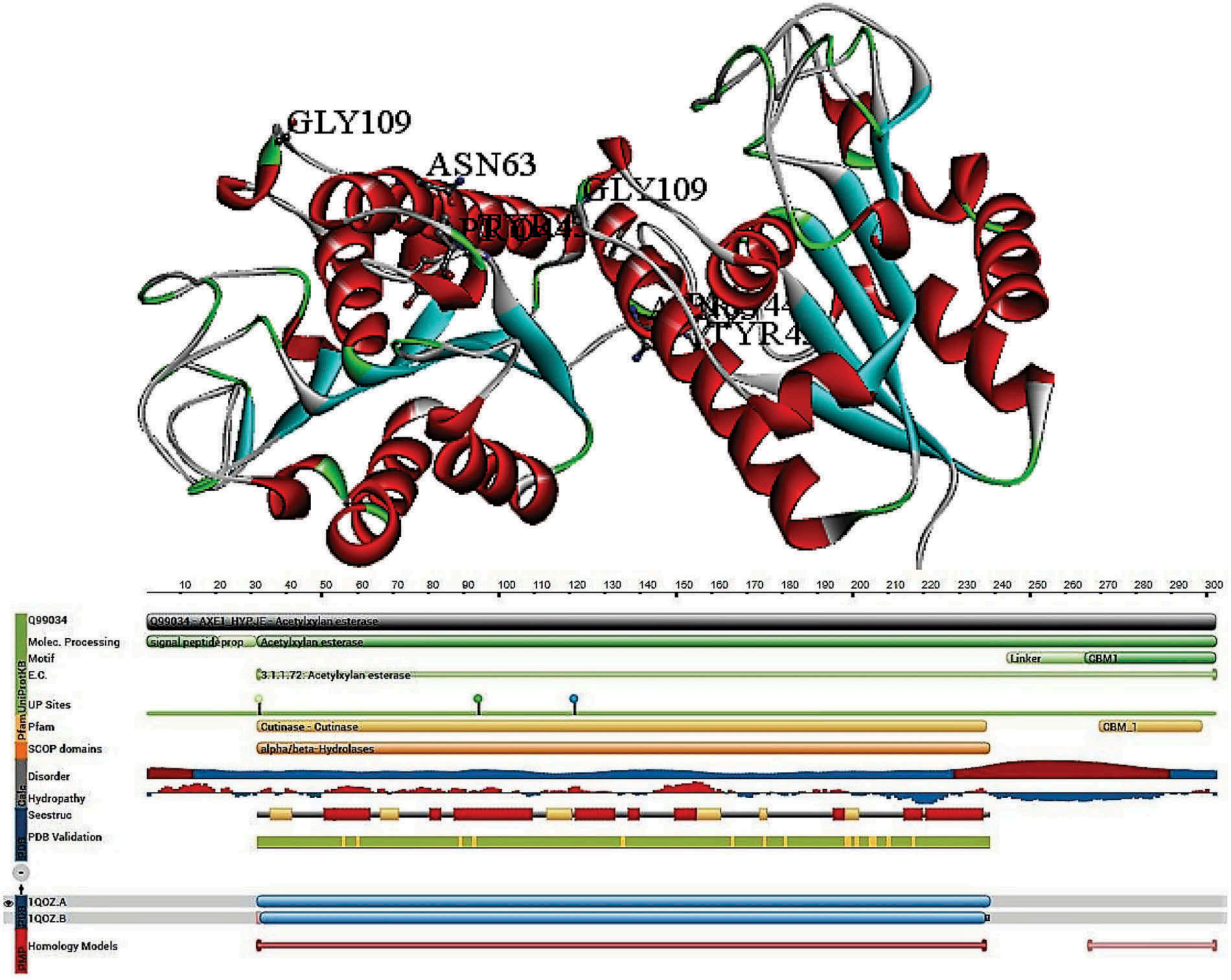
Structural properties of *Trichoderma reesei* acetyl xylan esterase (carbohydrate esterase class-5) (PDB ID:1QOZ), A) complete structure of AcXEII B) Topological view of AcXEII (Hakulinen et al. 2000).

The 3-D structure of *Trichoderma reesei* catalytic core domain was explained by Hakulinen et al. (2000), to reveal the deacetylation mechanisms employed by AcXE (Hakulinen et al. 2000). The 1.9 Å resolution of the catalytic core crystal structure contains α/β/α sandwich fold like that of the AcXEII and cutinase structures of *Penicillium purpurogenum* and *Fusarium solani*; all these enzymes belong to the CE-5 family with a common feature of α/β hydrolase fold. The complete protein dimensions of the AcXEII was found to be 45 × 35 X 30 Å, with six parallel β-strands (A1, 5–10; A2, 36 – 40; A3, 83–89; A4, 125–131; A5, 143–144; and A6,167–170) together forming the central β-strand. These β-strands are surrounded by four helices with two on either side H1, 23–32; H2, 57–78; H3, 91–10; and H6, 190–205, apart from these helices, it also contains two short and four 3_10_ helices situated at H4, 119–124 and H5, 184–188 and G1, 20–22; G2, 50–52; G3, 106–108; and G4, 164–166, respectively. There are total of 10 cysteine residues forming five disulphide bridges situated at Cys2–Cys79; Cys46–Cys52; Cys101–Cys161; Cys147–Cys179; and Cys171–Cys178. The narrow opening present near C-terminal end of A3, 83–89 (β-strand) containing the Ser^90^–His^187^–Asp^175^ catalytic triad forms the active site. The characteristic open α/β structure formed due to adjacent connections on the opposite sites of β-sheet. The cleft is formed due to the connections formed by A1 (one side of the sheet at loop 10 to 20) and A3 strand on either side (near catalytic residues of Ser^90^) with α-helices (H1 and H3) on opposite sides of β-sheets. The small helices and loops surrounding the cleft (12–14, 46–61, 105–109, 151–153,175–188) are majorly involved in binding to the substrate where especially aromatic amino acid residues Tyr^57^, Phe^152^ and Tyr^177^ play a crucial role. The *T. reesei* catalytic core domain (1QOZ) is a glycoprotein with Asn^63^ as N-glycosylation site, because of the presence of N-glucans, 1QOZ is represented as GlcNAc_2_Man _(1–6)_ P, which is clearly shown in the crystal structure of 1QOZ (Figure 4(A) and 4(B)) (Hakulinen et al. 2000).

To understand the catalytic mechanisms of AcXE, several studies were conducted with full and partially acetylated methyl glucopyranosides. Biely and his co-workers (Biely et al. 1996a, 1996b, 1997a) reported that AcXE *T. reesei* is capable of deacetylating at positions 2 and 3 with increased rate of hydrolysis due to the presence of free hydroxyl group on the other position. When a free hydroxyl group is present in the neighbouring position, a five-member transition state is formed where the acetyl group is loosely linked to both the positions 2 and 3. The catalytic triad (Ser^90^, His^187^ and Asp^175^) of AcXE plays a crucial role in deacetylation of xylan, and His^187^ residue removes a proton from Ser^90^ hydroxyl group (Hakulinen et al. 2000). The nucleophilic oxygen attacks carbonyl carbon of acetyl group forming a tetrahedral intermediate (oxyanion hole), which was stabilised by the hydrogen bonds. The hydrogen bonding stabilises the oxyanion (1QOZ) of the Thr13 (main chain) and Thr13 (side chain) stabilised by the Gln91. Earlier studies have reported that in many carbohydrate binding proteins, aromatic amino acid residues are involved in binding to the carbohydrate; similarly, the aromatic residues present in the active site of 1QOZ Tyr57, Phe152 and Tyr177 are involved packing against the xylose flat ring. It was reported that first xylose residue is packed against Tyr177 and the Phe152, and Tyr177 interacts with the next xylose residues of the xylan chain (Hakulinen et al. 2000). The first acetyl group fits into the active site of *T. reesei* 1QOZ; however, the second acetyl group does not interact with the active site as the Pro134, Phe152 and Tyr57 residues create several sterical restrictions. The structural orientation of the 2,3-di-*O-*acetyl-β-D-xylose proved the preference of AcXE towards monoacetylated xylan compounds rather than diacetylated xylan chains. In case of diacetylated xylan compounds, AcXE initially removes the first acetyl group of the compound and later this compound changes its orientation to fir the second acetyl group, thus one acetyl group is released by the AcXE of *T. reesei* followed by the second acetyl group. *T. reesei* AcXE are active on deacetylates of methyl-β-D-glucopyranosides and methyl-β-D-mannopyranosidases (Biely et al. 1997a). From the 3-D structure of 1QOZ, it is clear that acetyl groups of small side chains are readily removed than the acetyl groups of large side chains like 4-O-methylglucuronic acid and glucomannan (Hakulinen et al. 2000) (Figure 4).

### 2.6 *Bacillus pumilus* (CE class-7)

To understand the low paraxon sensitivity on *Bacillus pumilus* AcXE (BpAcXE) and also understand the effect of bound inhibitors on the conformational changes of the protein structure, Montoro-García et al. (2011) have conducted a X-ray crystallographic studies of BPAcXE in the absence of ligands (BpAcXE-apo) and in the presence of paraxon (BpcAXE-DEP) (Montoro-García et al. 2011). The X-ray crystallographic structures were determined at resolution 1.9Å (BpAcXE-apo) and 2.7Å (BpAcXE-DEP), respectively (Montoro-García et al. 2011). Previous studies have functionally characterised BPAcXE based on the sequence comparison (Vincent et al. 2003); enzyme activity (Martínez-Martínez et al. 2007) and molecular mass studies have proved that BPAcXE belongs to the AcXE/cephalosporin C deacetylases class enzymes. BPAcXE gene sequence showed sequence identity of 41%, 76% and 89% for AcXE encoding gene sequences of *Thermotoga maritima, B. subtilis and B. pumilus strain P213,* respectively (Vincent et al. 2003; Montoro-García et al. 2011).

**Figure 5.**
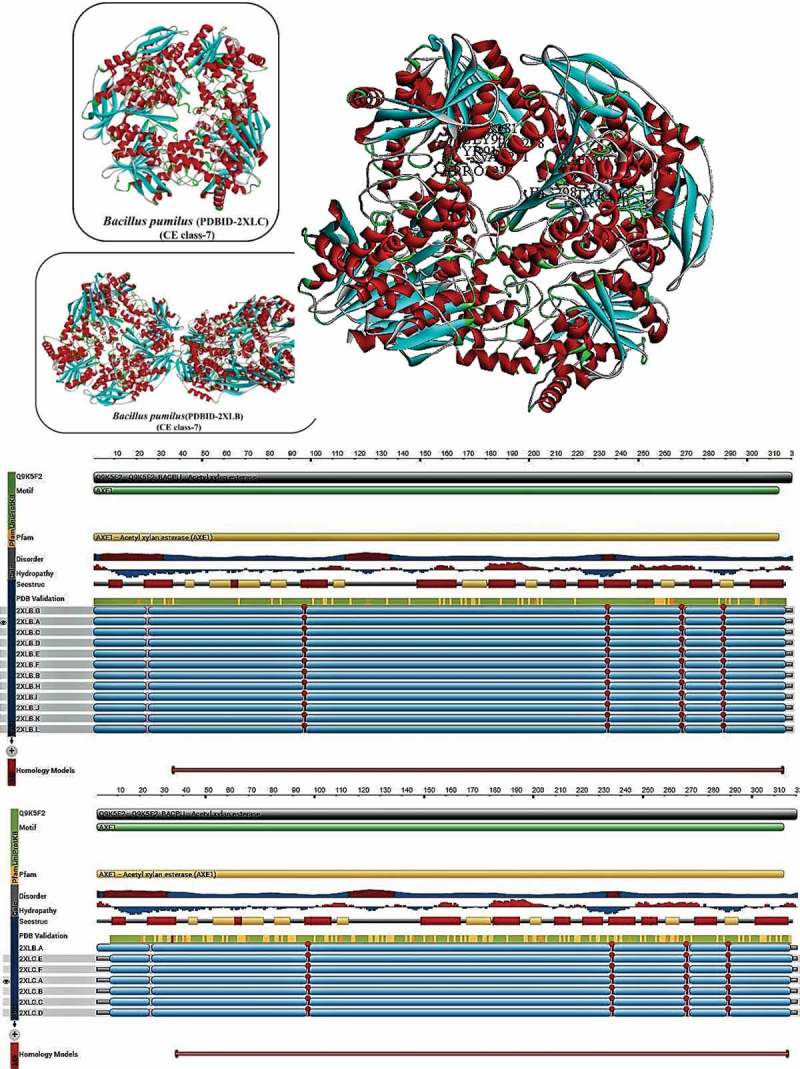
Structural properties of *Bacillus pumilus* acetyl xylan esterase (carbohydrate esterase class-7), A) complete structure of BPAcXE without ligands (PDB ID 2XLB); B) Topological view of BPAcXE without ligands C) complete structure of BPAcXE bound to Paraxon (PDB ID 2XLC) D) Topological view of BPAcXE bound to Paraxon (Montoro-García et al. 2011).

The AcXE from the above-mentioned organisms contain the CE class-7 conserved sequence motifs RGQ, GxSQG containing the serine residue and HE histidine residues for the catalytic triad formation (Vincent et al. 2003; Montoro-García et al. 2011). Both BpAcXE-apo and BpAcXE-DEP are homo-hexamers, which can be further classified as dimer of two trimers with overall structure resembling a doughnut. The 3-D structures of BpAcXE-apo and BpAcXE-DEP proteins closely resemble the structure of BsAcXE (*Bacillus subtilis* acetyl xylan esterase PDB ID: 1ODS (Vincent et al. 2003)) with rmsd values of 0.38 Å and 0.41Å from the superimposition of 317 and 311 Cα atoms, respectively. When compared to the well-known α/β hydrolases fold, the subunit folds of BpAcXE-apo and BpAcXE-DEP differs significantly in the presence of extra three-helix bundle, also the presence of second insertion containing two helices and one β-strand near N-terminus (Vincent et al. 2003). According to Montoro-García et al. (2011), three helix bundle insertion might play a crucial role in hexamer formation as this three-helix bundle interacts with one subunit of adjacent trimer and with another subunit from the same trimer, thus contacting the previous loop of α-helix (α11) on the C-terminal which also resides important His^298^ residue of the catalytic triad. These interactions and trimer formation of the 3-D structure might play a significant role in stabilising the active centre and the nearby substrate-binding gorge region of the protein. According to Vincent et al. (2003), the hexameric structures of these enzymes might relate to the limiting substrate size, which can be explained by the formation of a central chamber by six active centres accessed only through a central opening (Vincent et al. 2003). The occurrence of Asp^269^, His^298^ and Ser^181^ catalytic triad in the central chamber of the protein will support the strong specificity for small substrates by BPAcXE and BsAcXE enzymes. The BPAcXE crystals grown in the presence of paraxon showed a great mass density which is continuous with Ser^181^ Oγ and fits a diethyl phosphate (DEP) group (Montoro-García et al. 2011). The serine residue present in the catalytic site binds covalently to the paraxon moiety, which releases *p-*nitrophenyl moiety through oxyanion attack of catalytic serine residue. The DEP of BPAcXE is enclosed between Tyr91 of main chain, His289 of catalytic triad and Tyr206 phenolic ring, which is packed by the hydrophobic cushion formed by Tyr222, Phe210 and Leu207; thus, DEP interacts with theTyr^206^ N atom of Tyr^91^, His ^298^(Montoro-García et al. 2011). The superimposition studies of BpAcXE-apo and BpAcXE-DEP showed that both the enzymes are identical to each other with rmsd values for individual units was 0.20Å for 311 Cα atoms and for total hexameric structure was 0.35 Å for 1866 Cα atoms (Montoro-García et al. 2011) (Figure 5).

### 2.7 *Thermotoga maritima* (CE class-7)

Three dimensional structure and functional properties of *Thermotoga maritima* acetyl esterase (TM0077) was revealed in the year 2012 by Levisson et al (Levisson et al. 2012). TM0077 is active against a wide range of acetylated compounds, such as cephalosporin and several other short chain esters, optimal around 100°C and pH7.5 (Levisson et al. 2012). TM0077 is an intracellular enzyme with a molecular mass of 37kDa containing 325 amino acid with no predicted signal. The gene organisation analysis has shown that TM0077 gene co-localises with xylanase (TM0075) and β-xylosidase encoding genes. The complete structure of TM0077-SeMet (TM0077 seleno-methionine incorporated) and the native apo model of TM0077 consists of native hexamer where calcium ion of each monomer is bound to Lys22, Gly26 and Asp25 through a water molecule. The superimposition studies have revealed that of TM0077-native and TM0077-SeMet structures are nearly identical, with a rmsd value of 0.12 Å for 321 Cα atoms. Usually, TM0077 resembles the typical α/β hydrolase fold with eight stranded β-sheet surrounded by α-helices on either sides and a β2 antiparallel on another strand. However, occurrence of three-helix insertion after β6 strand with an extension on N-terminus differentiates TM0077 from the α/β hydrolase fold (Levisson et al. 2012).

Generally, in α/β hydrolase fold, insertions present after β6 or β7 strands support in maintaining the shape of the substrate-binding site and in the N-terminus two α-helices (αA_1_ and αA_2_) and antiparallel β-strand (β1) align to eight β-strands. The two α-helices αA and αB consist a short 3_10_-helix on the N-terminus, structurally the central β-sheet is surrounded by αA_−1_, αA_−2_, αB, αC, αD, αD_1_, αD_2_, αD_3_, αE and the 3_10_-helix η_2_ on one side and αA, αF and 3_10_-helix η_1_ on another side. The quaternary structure of the TM0077 consists of two hexamers, where each hexamer consists a dimer of trimers which are arranged side by side (Levisson et al. 2012). The analysis of the TM0077 hexameric structure showed two major interfaces, first interface is between subunits A and B, identical to C/D and E/F supported by seven hydrogen bonds on average, with a surface area 1024 Å^2^ contributed by each chain. The second interface is present between the A/F, B/C and D/E which is again stabilised by 17 hydrogen bonds with surface area 1079 Å^2^ contributed by each chain (Levisson et al. 2012).

The overall hexameric structure of the TM0077 resembles a doughnut shape, with the six active sites buried internally in the complex forming an oval-shaped cavity, which are accessed only through two entrances (each entrance is ~ 13 Å wide) on each side of the flat hexamer and connected to a tunnel ~ 10 Å for reaching to the inner cavity. The internal cavity of the entrances is blocked by three phenylalanine residues composing half of the hexamer. The active site of the TM0077 contains a standard catalytic triad with Ser188 (nucleophile and catalytic residue), His303 (protein acceptor/donor) and Asp274 (stabilises histidine) (Levisson et al. 2012). The Ser188 is situated in the conserved pentapeptide sequence of GGSQG, which is a signature sequence in esterases and lipases. The positions of catalytic triad residues Ser188, Asp274 and His303 are constant with their expected locations in the standard fold of α/β hydrolase family. Three glycine residues Gly186, Gly187 and Gly190 are arranged near the catalytic Ser188 residue facilitating contact to the nucleophile elbow, which prevents the steric hindrance for serine residue. The backbone amide groups of Tyr92 and Gln189 are required for the formation of oxyanion hole. The amino acid residues from the helices αA and αF are involved in bordering substrate binding site and the residues from β-strands 4,5,6, and the adjacent C-terminal loops are involved in forming a base. Thus, complete pocket attains a hydrophobic property though it contains polar amino acid residues (Gln88, Asp210 and Gln314), which might interact with substrate (Levisson et al. 2012).

**Figure 6.**
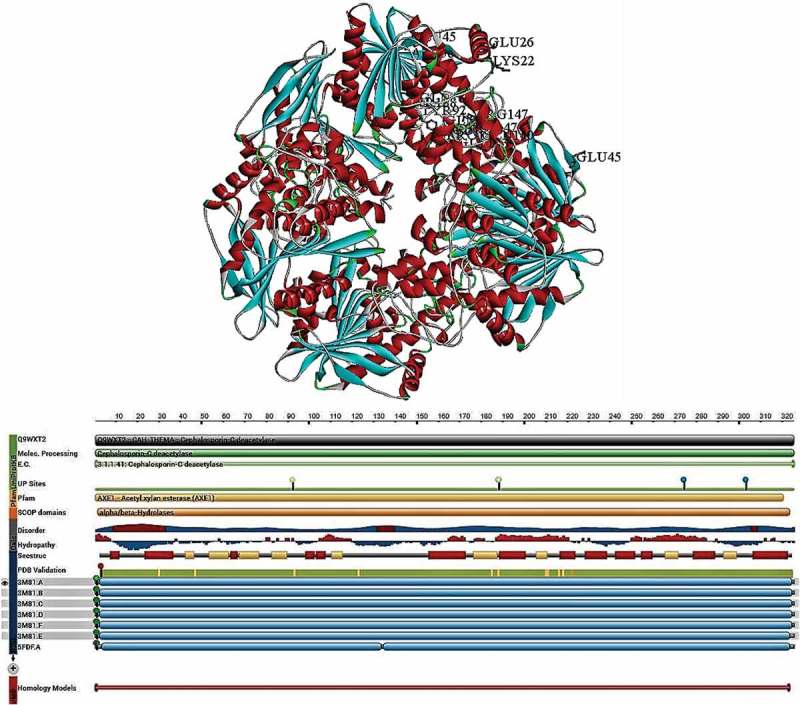
Structural properties of *Thermotoga maritima* acetyl xylan esterase (carbohydrate esterase class-5) (PDB ID:3M81), A) complete structure of AcXEII B) Topological view of AcXEII (Levisson et al. 2012).

The residue Ser188 is connected to imidazole group of His303 through hydrogen bond, extra density observed near Ser188 side chain was found to be chloride (based on its shape), electron density size and its geometry of interactions with the nearby residues. The chloride ion is bound at the oxyanion hole entrance through hydrogen bonds with backbone residues of Tyr92 and Gln189 (Levisson et al. 2012). The TM0077 was found to be active against short-chain ester groups (C2 and C3); in addition, it was also found to be active against glucose pentaacetate, but it was found to be inactive against acetylated xylan and xylan. Thus it can be classified as acetyl esterase. The protein is not secreted due to the lack of sequences coding for a specific signal, so the predicted intracellular location of TM0077 is consistent with its role other than deacetylating of extracellular xylan. Thus, biologically, TM0077 might be required for the removal of acetyl groups obtained through xylan degradation (deacylated xylose), which are imported into cytoplasm and form the substrates for the β-xylosidase, the clustering of TM0077 gene with genes involved in xylose metabolism also supports its proposed function. The TM0077 was studied for its positional specificity towards 4-nitrophenyl-β-D-xylopyranoside, and TM0077 specifically hydrolyses acetates present at 2, 3 and 4 positions of 4-nitrophenyl-β-D-xylopyranoside equally. Lack of specificity for the position of acetate groups provides access to a broad range of substrates for CE class-7 enzymes. The CE class-7 family esterases and deacetylases are active against substrates, such as acetylated xylooligosaccharides and antibiotic cephalosporin C. TM0077 exhibited ~ 10 higher activity compared with *Bacillus pumilus* AcXE against cephalosporin C and 7-aminocephalosporanic acid (7-ACA) (Levisson et al. 2012) (Figure 6).

The catalytic mechanism of esterases consists of two major reactions: first reaction involves nucleophilic attack by catalytic serine on carbonyl carbon atom of the substrate, forming an acyl-enzyme by liberating alcohol. Second reaction involves a water molecule which does nucleophilic attacks on the acyl-enzyme resulting in carboxylate by breaking acyl-enzyme bonds (Hedstrom 2002). Although the catalytic mechanism of esterases is well established, the strong interactions among the initial tetrahedral complex (intermediate) was supposed to collapse back to the reactant complex during the nucleophile attack of substrate (Hedstrom 2002). Previous studies have proposed an alternate catalytic mechanism for esterases, which prevents the collapse of tetrahedral intermediate during primary catalytic cycle, causing the residues to separate and further the reaction is carried out (Bizzozero and Dutler 1981; Ash et al. 2000; Hedstrom 2002). According to Mark et al., the native TM0077 and inhibitor-bound TM0077 proteins support the above-proposed mechanism (Levisson et al. 2012).

### 2.8 Geobacillus stearothermophilus

Structural and functional properties of monomeric and octameric *Geobacillus stearothermophilus* AcXE were revealed by Lansky et al. (2014). The AcXE2 complete protein structure resembles to the SGNH hydrolase fold and principally similar to α/β hydrolase fold differentiated by the location of the catalytic residues and the lack of nucleophilic elbow (Wei et al. 1995). The five-central parallel-β-sheet edged by two layers of helices can be classified into eight α-helices and five 3_10_ helices, altogether constituting to the fold. The positions of eight β-helices, five helices and 3_10_ helices are as follows: 8–13 (β1), 55–58 (β2), 84–88 (β3), 130–134 (β4), 168–170 (β5), 14–19 (α1), 36–49 (α2), 65–77 (α3), 90–100 (α4), 109–125 (α5), 145–166 (α6), 171–181 (α7) and 196–211 (α8) and 50–53 (h1), 78–81 (h2), 103–107 (h3), 126–128 (h4), and 184–188 (h5), respectively (Lansky et al. 2014). The structural positions of these eight β-helices display a topology of −1x + 2x + 1x + 1x, structurally this monomer can be divided into two parts convex and concave surfaces. The helices α3, α4, α5, α6, h2, h3 and h4 were arranged on the convex surface formed by β-sheets and α1, α2, α7, α8, h1 and h5 are situated on the concave surfaces. The dimeric asymmetric units of AcXE2 contains two AcXE2 chains named as A and B; the monomers constituting to the overall octameric structure were named as C and D, E and F, G and H, respectively, giving an overall appearance of a donut-shaped torus made up of two circular staggered tetramer layers located on top of each other. The tetramer I contains A, C, E and G chains and tetramer-II contains B, D, F and H chains, respectively. Majorly, three significant interactions, two hydrogen bonds (non-conserved) and a π-stacking interaction among the C-terminal region residues of the two protein chains forming the monomer–monomer contacts resulting in asymmetric dimer units. The π-stacking interaction was between two aromatic Trp215 residues of chains A and B, and the hydrogen bonds were formed between Arg205 (Chain A NH2 molecule) and Trp215 (chain B, O molecule) similarly Arg205 (chain B, NH2 molecule) and Trp215 (Chain A, O molecule). Four salt bridges between Glu105 OE1 and OE2 of one chain and Arg55 NH1 and NH2 of the other chain maintain the contact between the two tetramers. AcXE2 octamer contains eight polypeptide chains located around the central cavity, which is sectioned into four local hollows; these hollows are highly significant due to the presence of two active sites (belonging to different chains and different tetramers) separated by 5 Å, approximately (Lansky et al. 2014). Thus, AcXE2 enzyme contains four pairs of double catalytic sites located around the C4 axis of the octamer facing towards the central cavity. The complete octameric protein is about 100 Å in diameter with the diameter of cavity ranging up to 35 Å, resulting in a large pocket.

The active site of the AcXE2 contains Ser15, His194 and Asp191 constituting the standard catalytic triad of the serine proteases and esterase family proteins. The catalytic residues are well stabilised by the strong interactions formed by two hydrogen bonds (Ser15 OG to His194 NE2 and His194 ND1 to Asp191OD2). From the above interactions, the catalytic roles of Asp191 and His194 residues were confirmed to promote the nucleophilic attack on the deprotonated Ser15 side chains on the ester bonds of the substrate molecule, which constitutes for the first step of the hydrolysis. The structural roles of the catalytic residues were also supported by the site-directed mutagenesis experiments of the catalytic residues S15A, H194A and D191A, which were totally inactive (Alalouf et al. 2011). The structural homology with the other members of SGNH hydrolase family proteins suggests that residues involved in oxyanion hole formation of Gly63 and Asn92 (Alalouf et al. 2011). The crystal structures of AcXE2 also confirms the arrangement of these residue to maintain exact distance and appropriate orientation for interacting and stabilising the catalytic anionic intermediate (Lansky et al. 2014). The oligomeric catalytic site arrangement in AcXE2 deviates from the typical serine esterases, although the active site of each AcXE2 monomer follows as typical serine esterases. The presence of relatively strong interactions (hydrogen bonds and π-stacking interaction) plays a crucial role in supporting Asp191 and His194 in maintaining proper orientation with Ser15 residue. Interactions between Arg192 and Tyr184 and Trp190 and Trp95 bring the loop containing Asp191 and His194 near to Ser15 in correct position and conformation resulting in appropriate geometry of the Ser-Asp-His catalytic triad.

**Figure 7.**
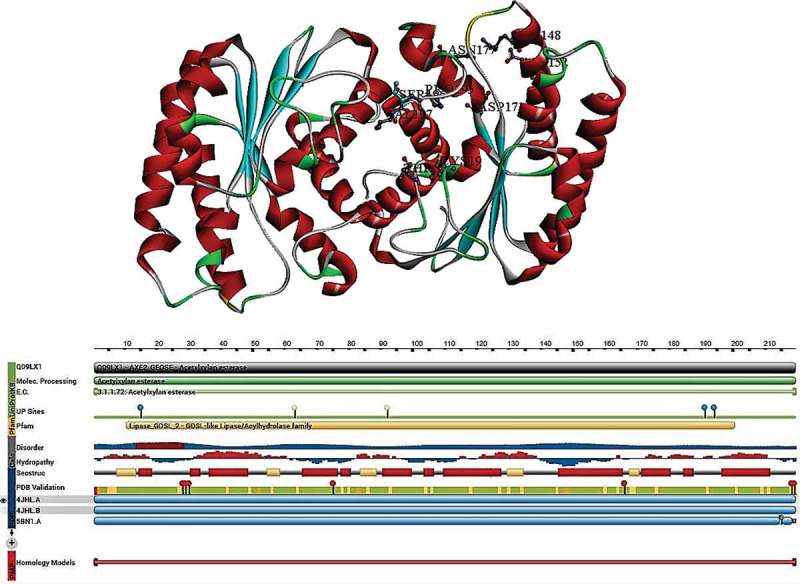
Structural properties of *Geobacillus stearothermophilus* acetyl xylan esterase (PDB ID: 4JHL), A) complete structure of AcXE2 B) Topological view of AcXE2 (Lansky et al. 2014).

According to Vincent et al. (2003), the reason behind the unique structure of AcXE2 was to protect the active sites from the usual cytoplasmic contents and guard the substrate from accidental and unregulated hydrolysis (Vincent et al. 2003). The arrangement of the AcXE2 active sites to the central cavity also suggests that catalytic residues are exposed only to selected contents present in the internal cavity of the torus octamer, which also suggests that the central cavity also stops larger substrates to enter the central catalytic space and reacting with the active sites (Vincent et al. 2003; Montoro-García et al. 2011). The arrangement of two catalytic sites forming the close-proximity to each other might provide several synergistic effects to the catalytic function. This type of assembly will accommodate the two active sites to act on the same substrate simultaneously (hemicellulose with several acetyl units) and increasing the rate of hydrolysis. Experiments conducted by Alalouf et al. (2011) have also confirmed the catalytic function of AcXE2 on 2,3,4-tri-O-acetyl methyl-β-D-xylopyranoside, where AcXE2 was able to deacetylate at positions 3 and 4 simultaneously (Alalouf et al. 2011). The octameric arrangement of AcXE2 is greatly stabilised by intermolecular interactions when compared to dimeric, tetrameric structures, thus enhancing the thermodynamic stability of the multimeric assembly (Lansky et al. 2014) (Figure 7) (Table 3).

**Table 3.** Structural and functional properties of acetyl xylan esterases belonging to different carbohydrate esterase classes.

PDB ID-CE-class	Structural Properties	Ref
*P. purpurogenum* **[1BS9]** **CE-1**	Reaction type: **Serine Esterase**, Catalytic amino acids: **Ser90-His187-Asp175**, Oxyanion hole: **Thr13-Gln91-Ser90**, Chain & length: **A, 207 residues**, SCOP and Pfam: **α/β hydrolases Cutinase like**, CATH: **3-layer α/β SRF**	(Ghosh et al. 2001)
*C. japonicus* [**2WAA**] **CE-2**	Reaction type: **Serine Esterase**, Catalytic amino acids: **Ser160-His335-Asp333**, Oxyanion hole: **Ser160-Gly205-Asn255**, Chain & length: **A, 347 residues**, SCOP and Pfam: **GDSL-like Lipase/Acylhydrolase family**, CATH: **3-layer α/β SRF**	(Montanier et al. 2009b)
*C. thermocellum* [**2C71]** **CE-3**	Reaction type: **Metalloenzymes, Aspartic esterases**, Catalytic amino acids: **Asp488-His539**, Oxyanion hole: **Asn508**, Chain & length: **A, 215 residues**, SCOP and Pfam: **GDSL-like Lipase/Acylhydrolase family**, CATH: **3-layer α/β SRF**	(Taylor et al. 2006)
*S. lividans* **[2CC0]** **CE-4**	Reaction type: **Metalloenzymes, Aspartic esterases**, Catalytic amino acids: **Asp13-His66**, Oxyanion hole: **Tyr103**, Chain & length: **A, B, 195 residues**, SCOP and Pfam: **7-stranded β/α Barrel, NodB polysaccharide deacetylase**, CATH: **α/β Barrel TIM Barrel**	(Taylor et al. 2006)
*T. reesei*, **[1QOZ]** **CE-5**	Reaction type: **Serine Esterase**, Catalytic amino acids: **Ser90-His187-Asp175**, Oxyanion hole: **Thr13 N-Thr13 Oγ**, Chain & length: **A, B, 207 residues**, SCOP and Pfam: **α/β hydrolases Cutinase like**, CATH: **3-layer α/β SRF**	(Hakulinen et al. 2000)
*B. pumilus*, **[2XLB]** **CE-7**	Reaction type: **Serine Esterase**, Catalytic amino acids: **Ser181-His298-Asp269**, Oxyanion hole: **Ser181**, Chain & length: **A, B, C, D, E, F, G, H, I, J, K, L 320 residues**, SCOP and Pfam: **α/β hydrolases, Acetyl xylan esterase 1**, CATH:**3-layer α/β SRF**	(Montoro-García et al. 2011)
*T. maritima* **[3M81]** **CE-7**	Reaction type: **Serine Esterase**, Catalytic amino acids: **Ser188-Asp274- His303**, Oxyanion hole: **Tyr92-Gln189**, Chain & length: **A, B, C, D, E, F. 337 residues**, SCOP and Pfam: **α/β hydrolases, Acetyl xylan esterase 1**, CATH: **3-layer α/β SRF**	(Levisson et al. 2012)
*G. stearothermophilus* **[4JHL]**	Reaction type: **SGNH-hydrolase**, Catalytic amino acids: **Ser15-His194-Asp191**, Oxyanion hole: **Gly63-Asn92**, Chain & length: **A, B 219 residues**, SCOP and Pfam: **GDSL-like Lipase/Acylhydrolase family**, CATH: **SGNH**	(Lansky et al. 2014)

### 2.9 Characterisation of acetyl xylan esterases

Several methods have been developed and used for the characterisation of AcXE conventionally at lab and industrial scales. Previous studies have reported that a wide range of chemical and natural substrates can be used for the quantitative assay of AcXE, such as a) birch hemicellulose (Biely et al. 1985); b) 4-nitrophenyl acetate (Biely et al. 1985); c) *p-*nitrophenyl acetate (Blum et al. 1999); d) α-naphthyl acetate (Degrassi et al. 1998); e) acetyl xylan (Degrassi et al. 1998); f) *N, N’-*diacetylchitobiose (Degrassi et al. 1998); g) galactose pentaacetate (Degrassi et al. 1998); h) cellulose acetate (Degrassi et al. 1998); i) 4-methylumbelliferyl acetate(Ding et al. 2007); and j) 7-amino cephalosporanic acid (Martínez-Martínez et al. 2007). Biely et al. (1985), which have proposed a method for the preparation of non-dialyzable fraction of acetylated and deacetylated xylan by steaming the birch wood at 200°C for about 10 min (to get non-cellulosic polysaccharide) and the deacetylation of the wood was performed by 0.2M NaOH for 16 h at 22 ^o^C, respectively (Biely et al. 1985).

- **Birch hemicellulose-based assay**: To the 20% (W/v) of birch hemicellulose (non-dialyzable portion), 0.4M phosphate buffer (pH 6.5) was added and thoroughly mixed. Equal proportions of diluted enzyme (in distilled water) was added to the reaction mixture and incubated. Stop the reaction by centrifugation or freezing, the supernatant was analysed for the acetic acid released using HPLC. One unit of AcXE can be defined by the amount of enzyme liberating 1 µmol acetic acid per every 10 mg of the substrate per 1 min (Biely et al. 1985).
- **4-nitrophenyl acetate assay**: To the freshly prepared saturated solution of 4-nitrophenyl acetate solution in 0.2 M phosphate buffer (pH 6.5), add 10 to 50 µl of enzyme extract solution. Incubate the reaction mixture at 22°C. Using a spectrophotometer, observe the released 4-nitrophenol from the reaction mixture at 410 nm, respectively. Enzyme activity can be calculated as “one unit of acetylesterase activity hydrolyses 1 µmol of the 4-nitrophenol acetate in 1 min” (Biely et al. 1985).
- ***p-*nitrophenyl acetate**: The *p-*nitrophenyl acetate assay can be performed using a 96-well microtiter plate. The reaction mixture consists of 10 mM phosphate buffer (pH 6.7), 200 µl aliquots of 100 µM *p*-nitrophenyl acetate and 10 to 50 µl of enzyme extract. The reaction is initiated by incubating the microtiter plate at 37°C. The reaction is continuously monitored for the change in absorbance at 405 nm using a microtiter plate reader. The *p-*nitrophenol acts as a standard (Blum et al. 1999).
- **Acetylated xylan and other substrates**: Degrassi et al. (1998) have reported an efficient acetylxylan esterase, which can be applied with different chemical and natural substrates, such as acetylated xylan, α-naphthyl acetate, *N, N’-*diacetylchitobiose, galactose pentaacetate, xylose tetra-acetate, glucose penta-acetate, *p-*nitrophenyl acetate and 4-methylumbelliferrylacetate, respectively. The reaction mixture consists of 500 µl of acetylated xylan (prepared by suspending 5% of acetylated xylan in 50mM sodium phosphate buffer with a pH7.0), 450 µl of 50mM sodium phosphate buffer (pH7.0) and up to 50 µl of pure enzyme extract. The reaction can be initiated by incubating at 37°C in an orbital shaker set at 150 rpm for 1 hour (Degrassi et al. 1998). However, when substrates such as acetyl xylan, *N, N’-*diacetylchitobiose, galactose pentaacetate, xylose tetra-acetate and glucose penta-acetate are used, a follow-up experiment should be performed to determine the acetate released using acetic acid assay kit (e.g. Biopharm kit). The enzyme activity can be defined as the one unit of enzyme that is required for liberating 1 µmol of *p-*nitrophenol/acetic acid per min, respectively. The reaction can be monitored at following absorbances, respectively, a) *p-*nitrophenol at 410 nm and b) 4-methylumbelliferone at 354 nm (Degrassi et al. 1998).
- **7-amino cephalosporanic acid assay**: Martínez-Martínez et al. (2007) have reported an AcXE assay using the substrate 7-amino cephalosporanic acid. The reaction can be carried using different aliquots of the enzyme extract ranging 25 µl, 50 µl, 75 µl, 100 µl, 125 µl and 150 µl, respectively. To the above reaction mixture, add 100 µl of the standard 7-amino cephalosporanic acid suspended in 5 mM phosphate buffer (pH 7.3). The reaction mixture was thoroughly mixed and monitored for the change in absorbance using a microplate reader set at 616 nm, respectively, for 10 min. Enzyme activity can be defined as “one unit of enzyme required for the formation of 1 µmol of acetic acid per minute under the standard reaction conditions” (Martínez-Martínez et al. 2007).

Due to their ability to exhibit a wide range of substrate and positional specificities, the enzyme activity of AcXE is quantitatively determined using a wide range of chemical- and natural-acetylated substrates. Previously reported studies have also determined the deacetylase activity of the microbial AcXE activities based on its deacetylating abilities of chemical and natural-acetylated substrates with different grade of acetylations, respectively (Shao and Wiegel 1995; Degrassi et al. 1998; Blum et al. 1999; Kosugi et al. 2002; Ding et al. 2007; Martínez-Martínez et al. 2007).

## 3. Industrial applications of acetyl xylan esterases

Industrially, esterases have wide range of applications; generally esterases are used in chemo or regioselectivity reactions, e.g. removal of ferulic acids from xylan and pectin (plant polysaccharides) by feruloyl esterase. Major applications of xylanase and acetyl xylan esterase lie in the paper-pulp (bleaching) and food processing, mainly in biofuel and biorefinery industries. Generally, the process of paper-making involves wide range of chemicals thus novel biotechnological methods were being developed to replace these chemicals. Biopulping is a fungal pretreatment method applied on woody plant biomass (lignocellulose) as prerequisite for the mechanical or chemical treatments. Mainly, fungal pretreatment is carried out for the removal of lignin subsequently from the woody biomass (Khonzue et al. 2011). The process includes debarking and chipping of wood chips followed by a brief steam explosion to minimise the natural load of microorganisms on the wood chips, after which the chips are cooled and inoculated with fungus. Biopulping is technically achievable process with low cost, less electricity usage, eco-friendly and higher mill throughputs for further mechanical pulping process. The major concern associated with the biopulping is the degradation of cellulose fibre eventually leading to low-quality paper. However, biopulping is highly efficient when compared to the chemical treatments. As the chemical pretreatments leave considerable amounts of lignin which later reprecipitates with cellulose fibers, giving the pulp its characteristic brown color which leaves considerable amounts of lignin which gets reprecipitated on to cellulose fibres, which gives the pulp its characteristic brown colour (Walia et al. 2015). Thus, the bio-bleaching method employing enzymes, such as hemicellulases (xylanases, AcXE) for improving the pulp quality, has gained higher attention in the last few years. The xylanases and AcXE used before chemical bleaching process are expected to cleave the linkages between the lignin and hemicellulose, which increases the availability of lignin to the bleaching chemicals and further extraction procedures. The major advantages of biobleaching are lesser usage of a) bleaching chemicals, b) organic halogen compounds, c) enhancing the pulp and paper quality, d) enhancing the pulp brightness and e) eco-friendly in nature (Azeri et al. 2010; Walia et al. 2015). AcXE and xylanases are highly used in the bioethanol (biofuel) industries for enhancing the breakdown and conversion of the plant cell-wall polysaccharides especially cellulose and hemicellulose. AcXE free the plan cell-wall polysaccharides from the ester linkages, which significantly reduces the catalytic properties of the xylanases and cellulases, respectively. Thus, the pretreatment of AcXE coupled with xylanases and cellulases will significantly enhance the downstream saccharification and fermentation steps, which are highly essential steps in the bioethanol production.

Last but not the least, deacetylation of 7-aminocephalosporanic acid is an industrially very important and highly studied process as it is involved in the production of industrially significant materials such as semi-synthetic β-lactam antibiotics, such as penicillins, cephalosporins, monobactams and carbapenems (Penicillins 2000; Ding et al. 2016). The β-lactam antibiotics are highly studied and commonly used group of antibiotics, which inhibit the cell-wall biosynthesis of bacteria. The 7-aminocephalosporanic acid is the major building material produced from the cephalosporin-c using the chemical and enzymatic processes. Apart from the above applications, xylanases and AcXE are highly applied in aiding the process of animal feed digestion as the lignocellulosic biomass is highly viscous. The pretreatment or supplementation of xylanases and AcXE enhances the release of macromolecules due to higher access of digestive enzymes to the substrate. Earlier studies have proved that supplementation of exogenous fibrolytic enzymes to animal feedstock has significantly improved the digestion of fibres (Beauchemin et al. 2003). Studies have also reported that addition of xylanases, AcXE and cellulases to the animal feed stock has significantly increased the average daily milk yield among the goats and Murray buffaloes, respectively (Bala et al. 2009; Shekhar et al. 2010).

## 4. Conclusions

The hemicellulose deacetylating and degrading enzymes are being highly studied in the recent years due to their major applications in saccharification of plant biomass. Previous studies have confirmed that glucuronoxylan (hardwood), galactoglucomannan (softwood) and arabinoxylans (annual plants) are highly or partially acetylated. However, application of modern powerful analytical methods can reveal the structural properties of plant cell walls and especially about the acetylation of plant polysaccharides. The present classification of carbohydrate deacetylases or esterases is based on the degree of similarities with amino acid homology and similarity in protein fold. Importantly, the AcXE classified in different classes of CE-classes exhibit different substrate and positional specificities. Thus, to establish a clear understanding about the structural and functional relationship of the AcXE enzymes studies must be conducted using specific substrates with selectively acetylated carbohydrates. Although it is difficult, it is possible to extract doubly acetylated xylose residues or acetyl group substituted xylose residues. However, it is difficult to isolate linear or monoacetylated carbohydrate residues from the plant cell-wall structures, as it was reported that acetyl groups migrate in aqueous mediums. Thus, the specifically acetylated substrates must be prepared by chemical approaches under waterless conditions. Synthesis of chemical derivatives of hexapyranosides or disaccharides, e.g. 4-nitrophenyl β-D-xylopyranoside, can significantly reveal the structural and functional relationships of carbohydrate deacetylases. Highly robust and reliable techniques such as nuclear magnetic resonance (NMR) spectroscopy can also be applied for understanding and revealing the structural and catalytic properties of AcXE’s. Further studies must be conducted to understand and reveal the catalytic potential, substrate and positional specificities of the CE. It is also important to conduct these studies as these enzymes exhibit great industrial and commercial applications, e.g. pharmaceutical, biofuel, biorefining, textiles and several other industrial applications.

## References

[CIT0001] Alalouf O, Balazs Y, Volkinshtein M, Grimpel Y, Shoham G, Shoham Y. 2011 A new family of carbohydrate esterases is represented by a GDSL hydrolase/acetylxylan esterase from Geobacillus stearothermophilus. Journal of Biological Chemistry. 286(49):41993–42001. 21994937 10.1074/jbc.M111.301051 PMC3234920

[CIT0002] Altaner C, Saake B, Tenkanen M, Eyzaguirre J, Faulds CB, Biely P, Viikari L, Siika-Aho M, Puls J 2003 Regioselective deacetylation of cellulose acetates by acetyl xylan esterases of different CE-families. Journal of Biotechnology. 105(1–2):95–104. 14511913 10.1016/s0168-1656(03)00187-1

[CIT0003] Ash EL, Sudmeier JL, Day RM, Vincent M, Torchilin EV, Haddad KC, Bradshaw EM, Sanford DG, Bachovchin WW 2000 Unusual 1H NMR chemical shifts support (His) Cɛ1—H⋅⋅⋅ O== C H-bond: proposal for reaction-driven ring flip mechanism in serine protease catalysis. Proceedings of the National Academy of Sciences. 97(19):10371–10376. 10.1073/pnas.97.19.10371 PMC27031 10984533

[CIT0004] Azeri C, Tamer U, Oskay M 2010 Thermoactive cellulase-free xylanase production from alkaliphilic Bacillus strains using various agro-residues and their potential in biobleaching of kraft pulp. African Journal of Biotechnology. 9(1): 063–072.

[CIT0005] Bacon JS, Gordon AH, Morris EJ, Farmer VC 1975 Acetyl groups in cell-wall preparations from higher plants. Biochemical Journal. 149(2):485–487. 1180909 10.1042/bj1490485 PMC1165644

[CIT0006] Bala P, Malik R, Srinivas B 2009 Effect of fortifying concentrate supplement with fibrolytic enzymes on nutrient utilization, milk yield and composition in lactating goats. Animal Science Journal. 80(3):265–272. 20163634 10.1111/j.1740-0929.2009.00634.x

[CIT0007] Beauchemin K, Colombatto D, Morgavi D, Yang W 2003 Use of exogenous fibrolytic enzymes to improve feed utilization by Ruminants 1 2. Journal of Animal Science. 81(14_suppl_2):E37–E47.

[CIT0008] Biely P 2012 Microbial carbohydrate esterases deacetylating plant polysaccharides. Biotechnology Advances. 30(6):1575–1588. 22580218 10.1016/j.biotechadv.2012.04.010

[CIT0009] Biely P, Côté G, Kremnický L, Greene R, Dupont C, Kluepfel D 1996a Substrate specificity and mode of action of acetylxylan esterase from Streptomyces lividans. FEBS Letters. 396(2–3):257–260. 8914998 10.1016/0014-5793(96)01080-0

[CIT0010] Biely P, Côté G, Kremnický L, Greene R, Tenkanen M 1997a Action of acetylxylan esterase from *Trichoderma reesei* on acetylated methyl glycosides. FEBS Letters. 420(2–3):121–124. 9459293 10.1016/s0014-5793(97)01500-7

[CIT0011] Biely P, Côté GL, Kremnický L, Weisleder D, Greene RV 1996b Substrate specificity of acetylxylan esterase from *Schizophyllum commune*: mode of action on acetylated carbohydrates. Biochimica Et Biophysica Acta (Bba)-Protein Structure and Molecular Enzymology. 1298(2):209–222. 8980647 10.1016/s0167-4838(96)00132-x

[CIT0012] Biely P, MacKenzie C, Puls J, Schneider H 1986 Cooperativity of esterases and xylanases in the enzymatic degradation of acetyl xylan. Nature Biotechnology. 4(8):731–733.

[CIT0013] Biely P, Mastihubová M, Puchart V 2007 The vicinal hydroxyl group is prerequisite for metal activation of *Clostridium thermocellum* acetylxylan esterase. Biochimica Et Biophysica Acta (Bba)-General Subjects. 1770(4):565–570. 17261352 10.1016/j.bbagen.2006.12.005

[CIT0014] Biely P, Puls J, Schneider H 1985 Acetyl xylan esterases in fungal cellulolytic systems. Febs Letters. 186(1):80–84.

[CIT0015] Biely P, Vršanská M, Tenkanen M, Kluepfel D 1997b Endo-β-1, 4-xylanase families: differences in catalytic properties. Journal of Biotechnology. 57(1–3):151–166. 9335171 10.1016/s0168-1656(97)00096-5

[CIT0016] Bitto E, Bingman CA, McCoy JG, Allard ST, Wesenberg GE, Phillips Jr GN 2005 The structure at 1.6 Å resolution of the protein product of the At4g34215 gene from *Arabidopsis thaliana*. Acta Crystallographica Section D: Biological Crystallography. 61(12):1655–1661. 16301800 10.1107/S0907444905034074

[CIT0017] Bizzozero S, Dutler H 1981 Stereochemical aspects of peptide hydrolysis catalysed by serine proteases of the chymotrypsin type. Bioorganic Chemistry. 10(1):46–62.

[CIT0018] Blair DE, Schüttelkopf AW, MacRae JI, Van Aalten DM 2005 Structure and metal-dependent mechanism of peptidoglycan deacetylase, a streptococcal virulence factor. Proceedings of the National Academy of Sciences. 102(43):15429–15434. 10.1073/pnas.0504339102 PMC1252587 16221761

[CIT0019] Blum DL, Li X-L, Chen H, Ljungdahl LG 1999 Characterization of an acetyl xylan esterase from the anaerobic fungus Orpinomyces sp. strain PC-2. Applied and Environmental Microbiology. 65(9):3990–3995. 10473406 10.1128/aem.65.9.3990-3995.1999 PMC99731

[CIT0020] Boraston AB, Bolam DN, Gilbert HJ, Davies GJ 2004 Carbohydrate-binding modules: fine-tuning polysaccharide recognition. Biochemical Journal. 382(3):769–781. 15214846 10.1042/BJ20040892 PMC1133952

[CIT0021] Carvalheiro F, Duarte LC, Gírio FM 2008 Hemicellulose biorefineries: a review on biomass pretreatments. Journal of Scientific & Industrial Research. 849–864.

[CIT0022] Carvalho AL, Dias FM, Prates JA, Nagy T, Gilbert HJ, Davies GJ, Ferreira LM, Romão MJ, Fontes CM 2003 Cellulosome assembly revealed by the crystal structure of the cohesin–dockerin complex. Proceedings of the National Academy of Sciences. 100(24):13809–13814. 10.1073/pnas.1936124100 PMC283503 14623971

[CIT0023] Caufrier F, Martinou A, Dupont C, Bouriotis V 2003 Carbohydrate esterase family 4 enzymes: substrate specificity. Carbohydrate Research. 338(7):687–692. 12644381 10.1016/s0008-6215(03)00002-8

[CIT0024] Chesson A, Gordon AH, Lomax JA 1983 Substituent groups linked by alkali-labile bonds to arabinose and xylose residues of legume, grass and cereal straw cell walls and their fate during digestion by rumen microorganisms. Journal of the Science of Food and Agriculture. 34(12):1330–1340.

[CIT0025] Correia MA, Prates JA, Brás J, Fontes CM, Newman JA, Lewis RJ, Gilbert HJ, Flint JE 2008 Crystal structure of a cellulosomal family 3 carbohydrate esterase from *Clostridium thermocellum* provides insights into the mechanism of substrate recognition. Journal of Molecular Biology. 379(1):64–72. 18436237 10.1016/j.jmb.2008.03.037

[CIT0026] Dalli SS, Rakshit SK 2015 Utilization of hemicellulose from lignocellulosic biomass-potential products In: Pittman KL, editor. Lignocellulose. New York: Nova Publishers Inc; p. 85–118.

[CIT0027] Dalrymple BP, Cybinski DH, Layton I, McSweeney CS, Xue G-P, Swadling YJ, Lowry JB 1997 Three *Neocallimastix patriciarum* esterases associated with the degradation of complex polysaccharides are members of a new family of hydrolases. Microbiology. 143(8):2605–2614. 9274014 10.1099/00221287-143-8-2605

[CIT0028] Degrassi G, Okeke BC, Bruschi CV, Venturi V 1998 Purification and characterization of an acetyl xylan esterase from *Bacillus pumilus*. Applied and Environmental Microbiology. 64(2):789–792. 10215579 10.1128/aem.64.2.789-792.1998 PMC106121

[CIT0029] Ding J-M, Yu-T-T, Han N-Y, Yu J-L, Li-J-J, Yang Y-J, Tang X-H, Xu B, Zhou J-P, Tang H-Z 2016 Identification and characterization of a new 7-aminocephalosporanic acid deacetylase from thermophilic bacterium *Alicyclobacillus tengchongensis*. Journal of Bacteriology. 198(2):311–320. 26527640 10.1128/JB.00471-15 PMC4751793

[CIT0030] Ding S, Cao J, Zhou R, Zheng F 2007 Molecular cloning, and characterization of a modular acetyl xylan esterase from the edible straw mushroom *Volvariella volvacea*. FEMS Microbiology Letters. 274(2):304–310. 17623028 10.1111/j.1574-6968.2007.00844.x

[CIT0031] Egana L, Gutierrez R, Caputo V, Peirano A, Steiner J, Eyzaguirre J 1996 Purification and characterization of two acetyl xylan esterases from *Penicillium purpurogenum*. Biotechnology and Applied Biochemistry. 24(1):33–99. 8756392

[CIT0032] Ghosh D, Erman M, Sawicki M, Lala P, Weeks DR, Li N, Pangborn W, Thiel DJ, Joernvall H, Gutierrez R 1999 Determination of a protein structure by iodination: the structure of iodinated acetylxylan esterase. Acta Crystallographica Section D: Biological Crystallography. 55(4):779–784. 10089308 10.1107/s0907444999000244

[CIT0033] Ghosh D, Sawicki M, Lala P, Erman M, Pangborn W, Eyzaguirre J, Gutiérrez R, Jörnvall H, Thiel DJ 2001 Multiple conformations of catalytic serine and histidine in acetylxylan esterase at 0.90 Å. Journal of Biological Chemistry. 276(14):11159–11166. 11134051 10.1074/jbc.M008831200

[CIT0034] Gille S, Pauly M 2007 O-acetylation of plant cell wall polysaccharides. Frontiers in plant science 3 (2012): 12. 10.3389/fpls.2012.00012 PMC3355586 22639638

[CIT0035] Grohmann K, Mitchell D, Himmel M, Dale B, Schroeder H 1989 The role of ester groups in resistance of plant cell wall polysaccharides to enzymatic hydrolysis. Applied Biochemistry and Biotechnology. 20(1):45–61.

[CIT0036] Hakulinen N, Tenkanen M, Rouvinen J 2000 Three-dimensional structure of the catalytic core of acetylxylan esterase from *Trichoderma reesei*: insights into the deacetylation mechanism. Journal of Structural Biology. 132(3):180–190. 11243887 10.1006/jsbi.2000.4318

[CIT0037] Hedstrom L 2002 Serine protease mechanism and specificity. Chemical Reviews. 102(12):4501–4524. 12475199 10.1021/cr000033x

[CIT0038] Hernick M, Fierke CA 2005 Zinc hydrolases: the mechanisms of zinc-dependent deacetylases. Archives of Biochemistry and Biophysics. 433(1):71–84. 15581567 10.1016/j.abb.2004.08.006

[CIT0039] Jacobs A, Lundqvist J, Stålbrand H, Tjerneld F, Dahlman O 2002 Characterization of water-soluble hemicelluloses from spruce and aspen employing SEC/MALDI mass spectroscopy. Carbohydrate Research. 337(8):711–717. 11950467 10.1016/s0008-6215(02)00054-x

[CIT0040] Khonzue P, Laothanachareon T, Rattanaphan N, Tinnasulanon P, Apawasin S, Paemanee A, Ruanglek V, Tanapongpipat S, Champreda V, Eurwilaichitr L 2011 Optimization of xylanase production from *Aspergillus niger* for biobleaching of eucalyptus pulp. Bioscience, Biotechnology, and Biochemistry. 75(6):1129–1134. 10.1271/bbb.110032 21670524

[CIT0041] Kibblewhite RP Brookes D 1976 Distribution of chemical components in the walls of kraft and bisulphite pulp fibres. Wood Science and Technology. 10(1):39-46.

[CIT0042] Kosugi A, Murashima K, Doi RH 2002 Xylanase and acetyl xylan esterase activities of XynA, a key subunit of the Clostridium cellulovorans cellulosome for xylan degradation. Applied and Environmental Microbiology. 68(12):6399–6402. 12450866 10.1128/AEM.68.12.6399-6402.2002 PMC134393

[CIT0043] Krastanova I, Guarnaccia C, Zahariev S, Degrassi G, Lamba D 2005 Heterologous expression, purification, crystallization, X-ray analysis and phasing of the acetyl xylan esterase from *Bacillus pumilus*. Biochimica Et Biophysica Acta (Bba)-Proteins and Proteomics. 1748(2):222–230. 15769599 10.1016/j.bbapap.2005.01.003

[CIT0044] Kremnický L, Mastihuba V, Côté GL 2004 Trichoderma reesei acetyl esterase catalyses transesterification in water. Journal of Molecular Catalysis B: Enzymatic. 30(5–6):229–239.

[CIT0045] Lansky S, Alalouf O, Solomon HV, Alhassid A, Govada L, Chayen NE, Belrhali H, Shoham Y, Shoham G 2014 A unique octameric structure of Axe2, an intracellular acetyl-xylooligosaccharide esterase from *Geobacillus stearothermophilus*. Acta Crystallographica Section D: Biological Crystallography. 70(2):261–278. 24531461 10.1107/S139900471302840X

[CIT0046] Levisson M, Han GW, Deller MC, Xu Q, Biely P, Hendriks S, Ten Eyck LF, Flensburg C, Roversi P, Miller MD 2012 Functional and structural characterization of a thermostable acetyl esterase from *Thermotoga maritima*. Proteins: Structure, Function, and Bioinformatics. 80(6):1545–1559. 10.1002/prot.24041 PMC3348966 22411095

[CIT0047] Lombard V, Ramulu HG, Drula E, Coutinho PM, Henrissat B 2014 The carbohydrate-active enzymes database (CAZy) in 2013. Nucleic Acids Research. 42(D1):D490–D495. 24270786 10.1093/nar/gkt1178 PMC3965031

[CIT0048] López-Cortés N, Reyes-Duarte D, Beloqui A, Polaina J, Ghazi I, Golyshina OV, Ballesteros A, Golyshin PN, Ferrer M 2007 Catalytic role of conserved HQGE motif in the CE6 carbohydrate esterase family. FEBS Letters. 581(24):4657–4662. 17826771 10.1016/j.febslet.2007.08.060

[CIT0049] Malunga LN, Beta T 2015 Antioxidant capacity of water-extractable arabinoxylan from commercial barley, wheat, and wheat fractions. Cereal Chemistry. 92(1):29–36.

[CIT0050] Martínez-Martínez I, Montoro-García S, Lozada-Ramírez JD, Á S-F, García-Carmona F 2007 A colorimetric assay for the determination of acetyl xylan esterase or cephalosporin C acetyl esterase activities using 7-amino cephalosporanic acid, cephalosporin C, or acetylated xylan as substrate. Analytical Biochemistry. 369(2):210–217. 17651681 10.1016/j.ab.2007.06.030

[CIT0051] Mastihubová M, Biely P 2004 Lipase-catalysed preparation of acetates of 4-nitrophenyl β-D-xylopyranoside and their use in kinetic studies of acetyl migration. Carbohydrate Research. 339(7):1353–1360. 15113674 10.1016/j.carres.2004.02.016

[CIT0052] Mitikka M Sorption of xylans on cellulose fibers. 1995. In Proceeding of the 8th International Symposium on Wood and Pulping Chemistry, June, Helsinki, Finland, 1995

[CIT0053] Montanier C, Money VA, Pires VM, Flint JE, Pinheiro BA, Goyal A, Prates JA, Izumi A, Stålbrand H, Morland C 2009a The active site of a carbohydrate esterase displays divergent catalytic and noncatalytic binding functions. PLoS Biol. 7(3):e1000071. 10.1371/journal.pbio.1000071 PMC2661963 19338387

[CIT0054] Montanier C, Money VA, Pires VM, Flint JE, Pinheiro BA, Goyal A, Prates JA, Izumi A, Stålbrand H, Morland C 2009b The active site of a carbohydrate esterase displays divergent catalytic and noncatalytic binding functions. PLoS Biology. 7(3):e1000071. 10.1371/journal.pbio.1000071 PMC2661963 19338387

[CIT0055] Montoro-García S, Gil-Ortiz F, García-Carmona F, Polo LM, Rubio V, Sánchez-Ferrer Á 2011 The crystal structure of the cephalosporin deacetylating enzyme acetyl xylan esterase bound to paraoxon explains the low sensitivity of this serine hydrolase to organophosphate inactivation. Biochemical Journal. 436(2):321–330. 21382014 10.1042/BJ20101859

[CIT0056] Pangborn W, Erman M, Li N, Burkhart BM, Pletnev VZ, Duax WL, Gutierrez R, Peirano A, Eyzaguirre J, Thiel DJ 1996 Characterization of crystals of *Penicillium purpurogenum* acetyl xylan esterase from high‐resolution x‐ray diffraction. Proteins Structure Function and Genetics. 24(4):523–524. 10.1002/(SICI)1097-0134(199604)24:4<523::AID-PROT13>3.0.CO;2-N 8860002

[CIT0057] Pauly M 1999 Development of analytical tools to study plant cell wall xyloglucan. Aachen: Shaker-Verlag.

[CIT0058] Pawar PM-A, Koutaniemi S, Tenkanen M, Mellerowicz EJ 2013 Acetylation of woody lignocellulose: significance and regulation. Frontiers in Plant Science. 4:118. 23734153 10.3389/fpls.2013.00118 PMC3659327

[CIT0059] Penicillins O 2000 Appropriate prescribing of oral beta-lactam antibiotics. Am Fam Physician. 62:611–620. 10950216

[CIT0060] Poutanen K, Rättö M, Puls J, Viikari L 1987 Evaluation of different microbial xylanolytic systems. Journal of Biotechnology. 6(1):49–60.

[CIT0061] Prates JA, Tarbouriech N, Charnock SJ, Fontes CM, LsM F, Davies GJ 2001 The structure of the feruloyl esterase module of xylanase 10B from Clostridium thermocellum provides insights into substrate recognition. Structure. 9(12):1183–1190. 11738044 10.1016/s0969-2126(01)00684-0

[CIT0062] Saba BC, Botbast RJ 1999 Enzymology of xylan degradation.In: Imam SH, Greene RV, Zaidi BR (eds) Biopolymers: utilizing natures advanced materials. American Chemical Society, Washington, D.C., pp 167–194.

[CIT0063] Scheller HV, Ulvskov P 2010 Hemicelluloses. Plant Biology. 61(1):263. 10.1146/annurev-arplant-042809-112315 20192742

[CIT0064] Schubot FD, Kataeva IA, Blum DL, Shah AK, Ljungdahl LG, Rose JP, Wang B-C 2001 Structural basis for the substrate specificity of the feruloyl esterase domain of the cellulosomal xylanase Z from Clostridium thermocellum. Biochemistry. 40(42):12524–12532. 11601976 10.1021/bi011391c

[CIT0065] Selig MJ, Adney WS, Himmel ME, Decker SR 2009 The impact of cell wall acetylation on corn stover hydrolysis by cellulolytic and xylanolytic enzymes. Cellulose. 16(4):711–722.

[CIT0066] Selig MJ, Knoshaug EP, Adney WS, Himmel ME, Decker SR 2008 Synergistic enhancement of cellobiohydrolase performance on pretreated corn stover by addition of xylanase and esterase activities. Bioresource Technology. 99(11):4997–5005. 18006303 10.1016/j.biortech.2007.09.064

[CIT0067] Shao W, Wiegel J 1995 Purification and characterization of two thermostable acetyl xylan esterases from Thermoanaerobacterium sp. strain JW/SL-YS485. Applied and Environmental Microbiology. 61(2):729–733. 7574610 10.1128/aem.61.2.729-733.1995 PMC167333

[CIT0068] Shekhar C, Thakur SS, Shelke SK 2010 Effect of exogenous fibrolytic enzymes supplementation on milk production and nutrient utilization in Murrah buffaloes. Tropical Animal Health and Production. 42(7):1465–1470. 20401691 10.1007/s11250-010-9578-2

[CIT0069] Taylor EJ, Gloster TM, Turkenburg JP, Vincent F, Brzozowski AM, Dupont C, Shareck F, Centeno MS, Prates JA, Puchart V 2006 Structure and activity of two metal ion-dependent acetylxylan esterases involved in plant cell wall degradation reveals a close similarity to peptidoglycan deacetylases. Journal of Biological Chemistry. 281(16):10968–10975. 16431911 10.1074/jbc.M513066200

[CIT0070] Teleman A, Lundqvist J, Tjerneld F, Stålbrand H, Dahlman O 2000 Characterization of acetylated 4-O-methylglucuronoxylan isolated from aspen employing 1 H and 13 C NMR spectroscopy. Carbohydrate Research. 329(4):807–815. 11125823 10.1016/s0008-6215(00)00249-4

[CIT0071] Teleman A, Nordström M, Tenkanen M, Jacobs A, Dahlman O 2003 Isolation and characterization of O-acetylated glucomannans from aspen and birch wood. Carbohydrate Research. 338(6):525–534. 12668108 10.1016/s0008-6215(02)00491-3

[CIT0072] Tenkanen M, Eyzaguirre J, Isoniemi R, Faulds CB, Biely P 2003 Comparison of catalytic properties of acetyl xylan esterases from three carbohydrate esterase families. ACS Publications.

[CIT0073] Topakas E, Kyriakopoulos S, Biely P, Hirsch J, Vafiadi C, Christakopoulos P 2010 Carbohydrate esterases of family 2 are 6‐O‐deacetylases. FEBS Letters. 584(3):543–548. 19968989 10.1016/j.febslet.2009.11.095

[CIT0074] Vincent F, Charnock SJ, Verschueren KH, Turkenburg JP, Scott DJ, Offen WA, Roberts S, Pell G, Gilbert HJ, Davies GJ 2003 Multifunctional xylooligosaccharide/cephalosporin C deacetylase revealed by the hexameric structure of the *Bacillus subtilis* enzyme at 1.9 Å resolution. Journal of Molecular Biology. 330(3):593–606. 12842474 10.1016/s0022-2836(03)00632-6

[CIT0075] Walia A, Mehta P, Guleria S, Shirkot CK 2015 Modification in the properties of paper by using cellulase-free xylanase produced from alkalophilic Cellulosimicrobium cellulans CKMX1 in biobleaching of wheat straw pulp. Canadian Journal of Microbiology. 61(9):671–681. 26220821 10.1139/cjm-2015-0178

[CIT0076] Weadge JT, Clarke AJ 2007 Neisseria gonorrheae O-acetylpeptidoglycan esterase, a serine esterase with a Ser-His-Asp catalytic triad. Biochemistry. 46(16):4932–4941. 17388571 10.1021/bi700254m

[CIT0077] Wei Y, Schottel JL, Derewenda U, Swenson L, Patkar S, Derewenda ZS 1995 A novel variant of the catalytic triad in the Streptomyces scabies esterase. Nature Structural & Molecular Biology. 2(3):218–223. 10.1038/nsb0395-218 7773790

[CIT0078] Willför S, Sundberg K, Tenkanen M Holmbom B 2008 Spruce-derived mannans–a potential raw material for hydrocolloids and novel advanced natural materials. Carbohydrate Polymers. 72(2):197-210.

[CIT0079] Wysokińska Z 2010 A market for starch, hemicellulose, cellulose, alginate, its salts and esters, and natural polymers, including chitin and chitosan: analysis results. FIBRES & TEXTILES in Eastern Europe. 18(6):83.

[CIT0080] Zhang J, Siika-Aho M, Tenkanen M, Viikari L 2011 The role of acetyl xylan esterase in the solubilization of xylan and enzymatic hydrolysis of wheat straw and giant reed. Biotechnology for Biofuels. 4(1):1. 22185437 10.1186/1754-6834-4-60 PMC3259036

